# Exploring the Impact of a High‐Fat Diet on Brain Homeostasis: A Comprehensive Analysis of the Absence of Inflammation

**DOI:** 10.1002/mnfr.70168

**Published:** 2025-07-28

**Authors:** Laura Plantera, Kerstin Immig, Anne‐Kristin Fritsche, Madlen Reinicke, Alon Zemer, Alon Monsonego, Uta Ceglarek, Ingo Bechmann

**Affiliations:** ^1^ Institute of Anatomy, Faculty of Medicine University of Leipzig Leipzig Germany; ^2^ Institute of Anatomy and Cell Biology Martin‐Luther‐University Halle‐Wittenberg Halle (Saale) Germany; ^3^ Institute of Laboratory Medicine Clinical Chemistry and Molecular Diagnostics, Faculty of Medicine University of Leipzig Leipzig Germany; ^4^ The Shraga Segal Department of Microbiology Immunology and Genetics, Faculty of Health Sciences & The School of Brain Sciences, and Cognition Ben‐Gurion University of the Negev Beer‐Sheva Israel

**Keywords:** Alzheimer`s disease, flow cytometry, microglia, obesity, phytosterols

## Abstract

Excessive fat consumption increases the risk of Alzheimer's disease (AD), potentially through diet‐induced neuroinflammation. Microglia, the brain's immune cells, are affected by obesity and diet. Phytosterols (PS), plant‐derived cholesterol‐like compounds, accumulate in the brain with age, and their content correlates with dietary intake. We hypothesize that the accumulation of PS modulates microglial activation and exerts anti‐inflammatory effects. We investigated the effects of a normal diet (ND), high‐fat diet (HFD), HFD with 2% PS (HFD+2% PS), and HFD with 4% PS (HFD+4% PS) on neuroinflammation in female and male C57BL/6J mice. Flow cytometry (FC) of microglia showed no significant regulation of pro‐ (IFN‐γ, IL‐1β, TNF‐α) and anti‐inflammatory (IL‐10) cytokines due to diet, but sex‐ and age‐dependent differences were observed. Immunofluorescence staining showed no TREM2 upregulation, indicating a lack of microglial activation in response to HFD. PS supplementation significantly reduced HFD‐induced weight gain, suggesting metabolic effects. Contrary to existing research, we found no evidence of HFD‐induced neuroinflammation or microglial activation. However, the reduction in weight gain with PS supplementation suggests potential metabolic benefits, which could have implications for the treatment of obesity. The potential effects on neuroinflammation remain unclear.

## Graphical abstract

Male and female mice were fed different diets for up to 24 weeks to study the effects of phytosterols (PS) on weight gain and brain immune cells. PS reduced weight gain in HFD‐fed mice. Microglia were isolated from brains and analyzed, revealing no diet‐driven inflammation changes, but differences based on sex and age.

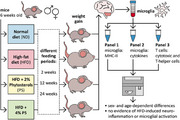

**Abbreviations**
AD: Alzheimer's disease
CNS: central nervous system
FC: flow cytometry
HFD: high‐fat diet
ND: normal diet
PGD2: prostaglandin D2
PS: phytosterols
TxB2: thromboxane B2
WHO: World Health Organization

## 1 Introduction

Obesity is an epidemic disease that confronts our society with challenges, especially in the Western world. It is a risk factor for chronic diseases such as cardiovascular disease, type 2 diabetes, certain cancers, and depression, but also for neurodegenerative diseases such as Alzheimer's disease (AD) [1]. The World Health Organization (WHO) defines obesity as an “abnormal or excessive fat accumulation that may impair health” and proclaims that “the fundamental cause of obesity and overweight is an energy imbalance between calories consumed and calories expended” (WHO, 9.6.2021). The worldwide prevalence of obesity nearly tripled between 1975 and 2016 and has now reached pandemic dimensions. In the WHO European Region, almost 60% of the adult population is now living with overweight or obesity (WHO European Regional Obesity Report 2022).

Microglia are long‐lived cells that exhibit aging characteristics over time [2]. They form the first line of the immune and defense system within the brain by continuously scanning the environment with their mobile projections [3]. They can quickly as well as effectively detect and respond to changes in the central nervous system (CNS), for example, due to infections. Therefore, their state of activation reflects neuroinflammation [4]. In addition to defense functions, microglial cells play important roles in maintaining homeostasis in the CNS [5, 6, 7]. Thus, they are involved in normal memory functions by being able to eliminate connections between neurons and thus form the basis for learning processes. By upregulating phagocytic functions, altering morphology, and expressing a range of bioactive effector molecules, microglia can respond specifically to pathological changes [8, 9].

Unlike other organs, the brain lacks a lymphatic drainage system for detoxification, so well‐defined (β‐amyloid) and poorly‐defined substances (corpora amylacea and autofluorescent [lipo] pigments such as lipofuscin) accumulate there during aging [10, 11, 12]. In addition to the perivascular drainage of molecules such as β‐amyloid from the neuropil into the subarachnoid space [12, 13], there is a second detoxification process of the brain through microglial cells that ensures and enables neuroprotection, synaptic plasticity, and learning processes through phagocytosis of extracellular toxins such as iron, soluble β‐amyloid, and synaptic remnants [14, 15, 16, 17].

Obesity is often characterized by chronic low‐level inflammation [18] and has been associated with increased inflammatory cytokine levels released by microglia in the murine system [8, 9, 18]. In fact, chronic activation and excessive phagocytosis might cause microglial senescence observed in the aged human brain, which we regard as a form of immune exhaustion [4, 19, 20, 21, 22, 23]. The term “neuroinflammation” is widely used, and its definition varies among authors [24].

The pancreas is an endocrine, diet‐sensitive organ. Therefore, we have chosen the pancreas to compare the diet‐derived response of lymphoid and myeloid cells in the periphery with the brain. The pancreas consists of an exocrine (acinar glands) and an endocrine component (islets of Langerhans). Pancreatic islets of Langerhans are affected by low‐grade tissue inflammation triggered by obesity [25]. This appears to be one of the main causes of insulin resistance [26]. The accumulation of macrophages, dendritic cells, and lymphocytes in islet cell inflammation due to long‐term obesity has been reported several times [27, 28, 29]. Furthermore, elevated levels of inflammatory cytokines and chemokines play critical roles in the progression of pancreatic *β*‐cell dysfunction [30, 31].

Phytosterols (PS), similar in structure to cholesterol, exclusively derive from plant food. Upon eating, they are carried in the circulation in serum lipoproteins together with cholesterol and—as we have shown previously—accumulate in the brain [32]. The accumulation of certain nutrition‐derived fatty acids, biologically active lipids, and their metabolites in the brain could influence aging and microglial cell loss [33, 34, 35]. Interestingly, concentrations of COX‐2‐derived lipid mediators such as prostaglandin D2 (PGD2) and thromboxane B2 (TxB2) inversely correlate with PS concentrations, suggesting an anti‐inflammatory effect of nutrition‐derived PS [32]. It is known that polyunsaturated fatty acids also have an influence on the structure of cell membranes and their signal transmission [36]. There is preliminary evidence that PS are incorporated into lipid‐rich domains of cell membranes, known as lipid rafts, and influence inflammatory signaling [37, 38].

In this study, we investigated the effects of normal diet (ND), high‐fat diet (HFD), high‐fat diet with 2% phytosterols (HFD+2% PS), and high‐fat diet with 4% phytosterols (HFD+4% PS) in female and male C57BL/6J mice on indicators of neuroinflammation using flow cytometry (FC) after different periods of feeding (2, 12, and 24 weeks) and immunofluorescence stainings of microglia.

## 2 Experimental Section

### 2.1 Animals and Diets

The experiments were performed using female and male wild‐type C57BL/6J mice, which were kept in the local animal facility under standard conditions as follows: 12 h dark/light cycle, group‐housed with free access to water and food. The animals were kept in groups of three in a species‐appropriate manner with objects, nesting material, and retreat areas. The welfare of the animals was controlled at least once a day by direct visual inspection and recorded in a score sheet. In order to avoid pain, suffering, and harm to the animals that were incompatible with the objective of the experiment, clear termination criteria were determined for the experimental project. Before tissue removal, all mice were transferred to the institute by an animal transport service. Throughout the whole experiment, it was ensured that no avoidable pain, suffering, or harm was inflicted on the mice.

Young adult mice (6 weeks [wk] old) were fed with an ND (9 kcal% fat, 67 kcal% carbohydrates, 24 kcal% protein; V1534‐300, ssniff Spezialdiäten, Soest, Germany) or an HFD (59 kcal% fat, 26 kcal% carbohydrates, 15 kcal% protein; E15772‐34, ssniff Spezialdiäten GmbH, Soest, Germany) with and without physiological doses of PS (BTC Europe: Vegapure 867 GN) for 2, 12, and 24 weeks. The diet composition of the HFD, PS, and a PS‐determination of our fodder samples by mass spectrometry are provided in the Supporting Information S1. Laboratory animals were divided into the following 24 groups (Table 1).

**TABLE 1:** *n*‐Numbers of the experimental design for FC of male and female mice.

	Male			Female			
	2 weeks	12 weeks	24 weeks	2 weeks	12 weeks	24 weeks	Sum ∑
**ND**	9	12	12	9	12	12	66
**HFD**	9	11	12	9	11	12	64
**HFD+2% PS**	9	12	12	9	12	12	66
**HFD+4% PS**	9	12	11	9	12	12	65
**Sum ∑**	36	47	47	36	47	48	**261**

Body weight and blood sugar were measured weekly during the whole experiment. All animal experiments were approved by the local state and university authorities. We performed this study in accordance with the guidelines of the Animal Experimental Committee following the German Animal Welfare Act as well as the European guidelines (Directive 2010/63/EU) concerning the protection of laboratory animals. All experimental procedures and protocols were authorized by the local ethics committee of the state of Saxony (Landesdirektion Sachsen, Leipzig, Germany, approval no. TVV 55/21).

### 2.2 Dosage Information/Dosage Regimen

PS from BTC Europe (Vegapure 867 GN) were supplied to ssniff Spezialdiäten, Soest, Germany, for incorporation into HFD feed pellets (ssniff, E15772‐34) at the specified concentrations. Vegapure is a commercial food supplement widely used in the food industry, which is also available in cardio capsules aimed at reducing cholesterol levels. The composition of the HFD, along with the technical data sheet and specifications for Vegapure, is provided in Supporting Information S1. Vegapure contains 67%–80% beta‐sitosterol and < 15% campesterol. The PS content in the feed pellets was validated by mass spectrometry and is shown for campesterol, sitosterol, and stigmasterol in Supporting Information S1.

Mice ingested PS through their diet, with free access to food throughout the experiment, which lasted 2, 12, and 24 weeks. The PS doses reflect human physiological doses, which are achievable through a regular diet. We supplemented 2% and 4% PS to the HFD, based on a publication by Cedó et al. in which a Western‐type diet has been supplemented with 2% PS composed of 80% beta‐sitosterol and 7% campesterol [39]. On average, female mice consumed 80 mg (HFD+2% PS) to 160 mg (HFD+4% PS) PS/day per 0.232 kg body weight, while male mice consumed 100 mg (HFD+2% PS) to 200 mg (HFD+4% PS) of PS/day per 0.305 kg body weight. For humans, this corresponds to 27.25 mg/kg/day (2% PS) and 54.5 mg/kg/day (4% PS), equivalent to 1.9–3.8 g/day for a 70 kg person. This range covers both lower and higher doses within the current recommended daily intake of 3 g/day in most countries [40].

### 2.3 Isolation of Microglia

At the end of the feeding experiments, mice were anesthetized with isoflurane (Baxter GmbH, Unterschleißheim, Germany) and transcardially perfused with ice‐cold PBS (Gibco, Life Technologies, Darmstadt, Germany) to clear the intravascular compartment of the brain from remaining blood cells. Brains were removed and transferred into ice‐cold PBS. Microglia cells were isolated as described by Immig et al. [41]. The isolated brains were minced and homogenized using a glass potter (Novodirect, Kehl, Germany), followed by trituration of fire‐polished Pasteur pipettes with decreasing diameters. Cell suspension was filtered through a 70 µm cell strainer (BD Falcon, BD Biosciences, Heidelberg, Germany) followed by a careful centrifugation (Labofuge 400R, Heraeus Instruments) at 900 rpm at 4°C for 10 min. Pellets were resuspended in 10 mL of 75% Percoll solution (GE Healthcare, München, Germany) covered with 10 mL of 25% Percoll solution. Finally, 6 mL of PBS was carefully added on top. The cell gradient was centrifuged at 1800 rpm at 4°C for 30 min. After centrifugation, the myelin‐containing layer was carefully removed. Mononuclear brain cells were collected from the thin layer between the 25% and 75% Percoll layer. Mononuclear cells were discriminated from lymphocytes by granularity, size, and for specific cell markers (see below flow cytometry staining) [41].

The isolation of pancreatic leucocytes is provided in the Supporting Information S2.

### 2.4 Flow Cytometry Staining

Cells were pre‐incubated with anti‐CD16/32 antibody (1:100, eBioscience, San Diego, California, USA) for 5 min to minimize unspecific binding of antibodies on Fc‐receptors [41]. Next, cells were incubated for 30 min with primary labeled antibodies on ice in the dark. **Panel 1**: CD45‐eFluor780 (1:100, Thermo Fisher); CD11b‐Pacific Blue (1:100, eBioscience); P2RY12‐PE (1:100, eBioscience); GR1‐AF647 (1:200, eBioscience); and MHC‐II‐PE‐Cy7 (1:125, eBioscience). **Panel 2**: CD45‐eFluor780 (1:100, Thermo Fisher); CD11b‐Pacific Blue (1:100, eBioscience); and P2RY12‐PE (1:100, eBioscience). **Panel 3**: CD45‐eFluor780 (1:100, Thermo Fisher); CD11b‐Pacific Blue (1:100, eBioscience); CD3‐PE‐Cy7 (1:100, eBioscience); CD4‐PE (1:100, eBioscience); and CD8‐AF647 (1:100, eBioscience). Afterward, all samples were labeled with live/dead dye (zombie UV Fixable Viability Kit, eBioscience) for 20 min at room temperature. Cells were washed with wash buffer (BD Perm/Wash Buffer, eBioscience) and centrifuged for 6 min at 1100 rpm at 4°C. The supernatant was discarded, and cells were fixed with 100 µL fixation/permeabilization solution (eBioscience) for 20 min on ice. Cells were washed with wash buffer and centrifuged for 5 min at 1200 rpm at 4°C. The supernatant was discarded, and samples of Panels 1 and 3 were rinsed in 200 µL wash buffer. To stain for intracellular cytokines in Panel 2, fixed/permeabilized cells were resuspended in 50 µL of wash buffer containing TNF‐α‐FITC (1:100, Thermo Fisher); IL‐1β‐PE‐Cy7 (1:100, Thermo Fisher); IL‐10‐APC (1:100, Thermo Fisher); IFN‐γ‐PerCP‐Cy5.5 (1:100, Thermo Fisher); and incubated on ice for 30 min in the dark. Cells were washed once again with wash buffer and centrifuged for 5 min at 1200 rpm at 4°C. The supernatant was discarded, and samples of Panel 2 were also rinsed in 200 µL wash buffer. For all FC stainings, gates were set accordingly to blank, and isotype controls were used in each experiment. Pancreatic lymphocytes were stained with the same antibodies as microglial lymphocytes. Details of the FC stainings of pancreatic leucocytes are provided in the supporting information, as well as all gating strategies (Supporting Information S2).

### 2.5 Statistical Analyses

Flow cytometry stainings (percentages and MFIs) were analyzed using FlowJo (FlowJo 10.8.1). Data from at least three independent experiments were evaluated by ordinary one‐way ANOVA or two‐way ANOVA and Tukey's multiple comparisons test. They were presented as the standard error of the mean (SEM) using GraphPad Prism (Prism 9.5.1). *p* values < 0.05 were considered to be statistically significant (*p < 0.05, **p < 0.01, and ***p < 0.001).

### 2.6 Fluorescence Labeling

Immunofluorescence stainings with anti‐IBA1 (ionized calcium‐binding adaptor molecule 1; Synaptic Systems, Göttingen, Germany) to label microglia and anti‐TREM2 (Triggering Receptor Expressed by Myeloid cells, R&D Systems, Inc., Minneapolis, USA) were performed based on a previously published protocol [35]. Perfused and fixed mouse brains were sliced into 50‐µm‐thick coronal floating sections using a vibratome (Leica VT 1200, Leica Biosystems, Wetzlar, Germany). After three washing steps with 0.3% Triton X‐100 in PBS for 10 min each time, slices were blocked for 1 h in PBS blocking buffer containing 5% normal goat serum (NGS) and 0.3% Triton X‐100 at room temperature. Afterward, coronal brain sections were incubated with the primary antibodies IBA1 (1:500) and TREM2 (1:50) diluted in PBS with 1% of NGS. Incubation was done overnight at 4°C. The next day, slices were rinsed three times with 0.3% Triton X‐100 in PBS and incubated with the secondary antibodies goat anti‐guinea pig Alexa Fluor 488 (1:200) (Thermo Fisher Scientific, Waltham, Massachusetts, USA) and goat anti‐sheep Alexa Fluor 647 (1:200) (Thermo Fisher Scientific) for 2 h at room temperature. Thereafter, sections were washed with PBS, stained for 5 min with 40, 6‐diamidino‐2‐phenylindole (DAPI; Thermo Fisher Scientific), and thoroughly rinsed in PBS. Finally, brain sections were mounted onto microscope slides and covered with Fluorescence Mounting Medium (DAKO, Agilent, Santa Clara, California, USA) and coverslips. For negative controls, the omission of primary antibodies, under otherwise identical conditions, resulted in the absence of any labeling [35].

### 2.7 Image Acquisition and Quantification of Fluorescence Staining

Microscopic images of IBA1 and TREM2 stainings were captured with a confocal microscope (LSM 700, Zeiss, Jena, Germany) applying a 40× /0.65 NA objective at constant exposure times within hypothalamic, hippocampal, and neocortical regions. Confocal z‐stack images were acquired using the ZEN 2 (blue edition) software (Zeiss) and an interval size of 2.0 µm for a total range of 30 µm [35]. Fluorescence intensity and staining area measurements of z‐stack maximum intensity projections were processed using ImageJ software (National Institutes of Health, Bethesda, Maryland, USA).

## 3 Results

### 3.1 Weight and Blood Sugar Data

**FIGURE 1:**
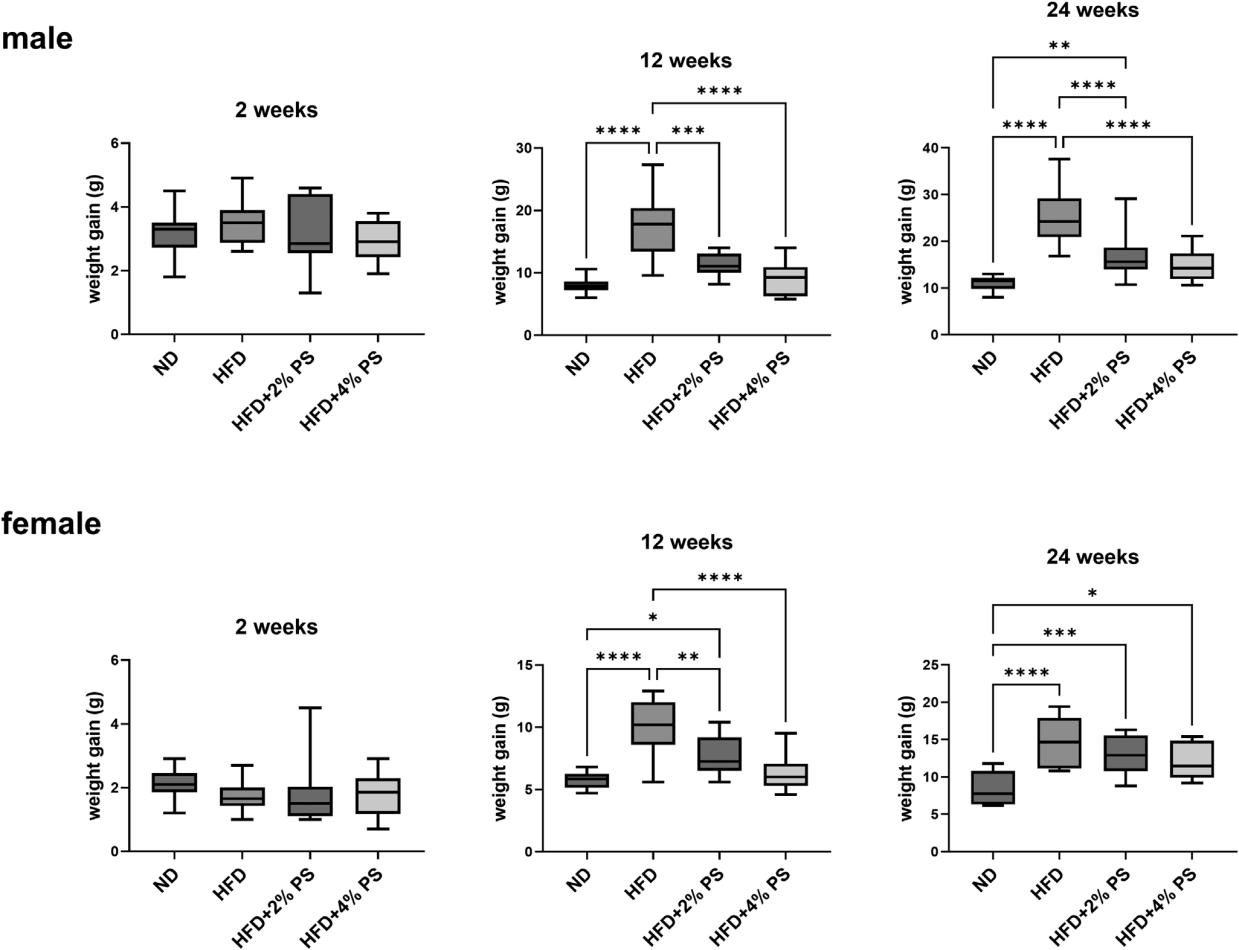
Weight gain. Weight gain due to ND, HFD, HFD+2% PS, HFD+4% PS after 2, 12, and 24 weeks of diet in male and female mice (one‐way ANOVA; *p* value ≤ 0.05).

**FIGURE 2:**
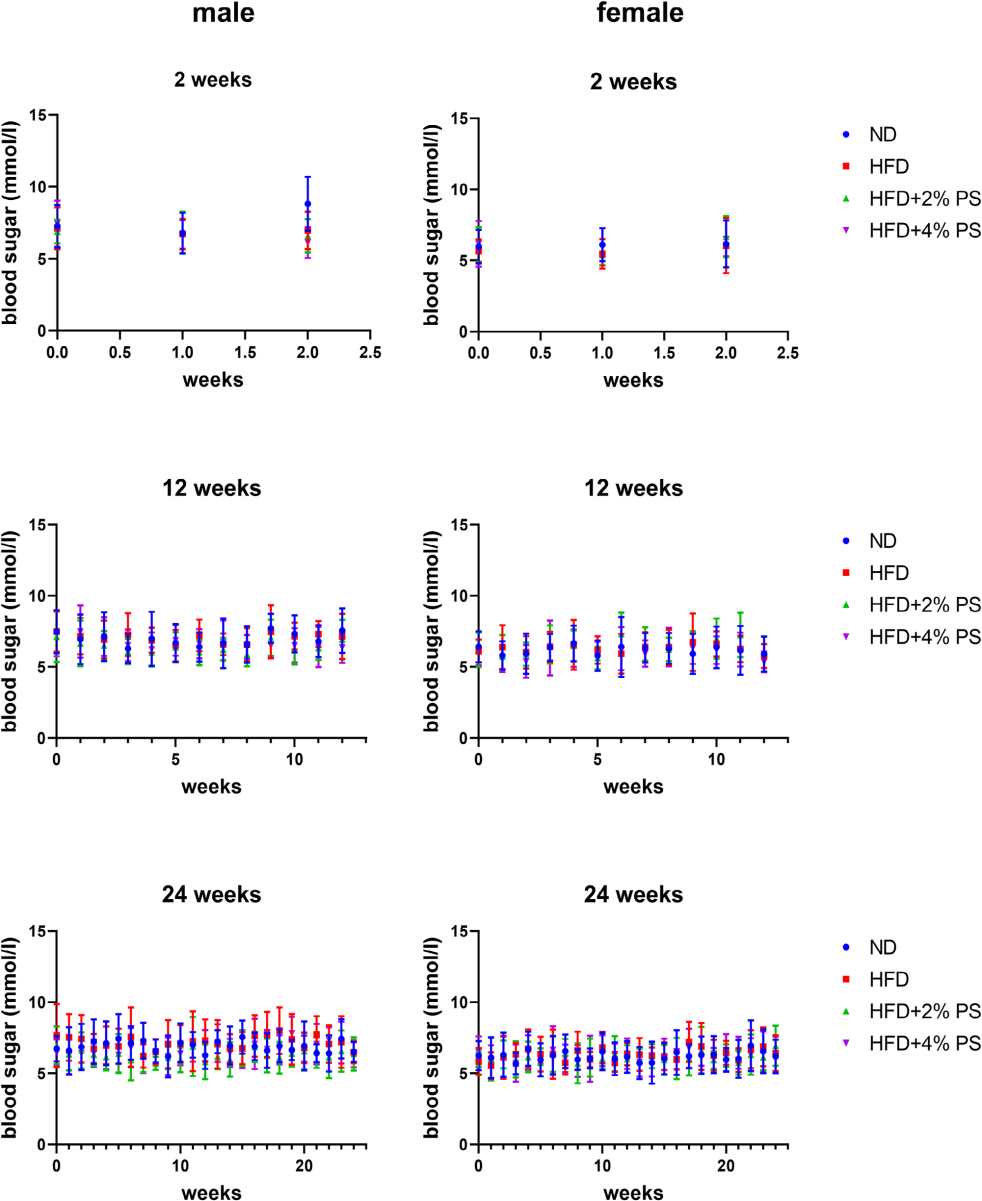
Blood sugar. Blood sugar levels of male and female mice fed with ND, HFD, HFD+2% PS, and HFD+4% PS for 2, 12, and 24 weeks (one‐way ANOVA; SD; *p* value ≤ 0.05).

A significantly reduced weight gain was observed in mice receiving HFD with PS compared to HFD only after 12 and 24 weeks in male as well as in female mice (Figure 1). The blood sugar was not impaired by the different diets after all three time periods (Figure 2).

### 3.2 Flow Cytometry

**FIGURE 3:**
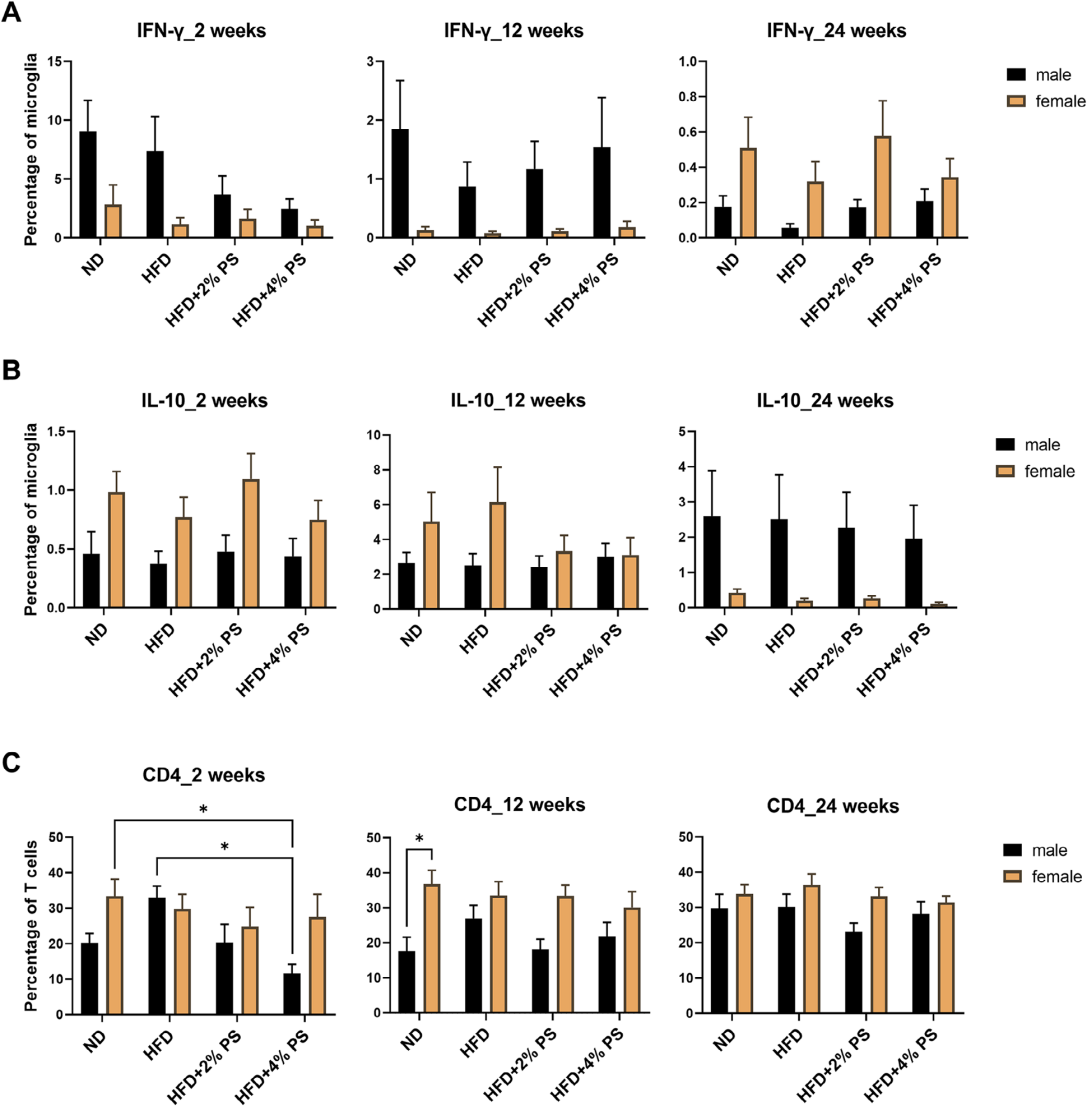
Brain—comparison between male and female mice. Microglia Panel 2 (**A**) IFN‐γ, (**B**) IL‐10, and brain lymphocytes Panel 3 (**C**) CD4‐positive T cells after ND, HFD, HFD+2% PS, HFD+4% PS after 2, 12, and 24 weeks (two‐way ANOVA; SEM; *p*‐value ≤ 0.05).

**FIGURE 4:**
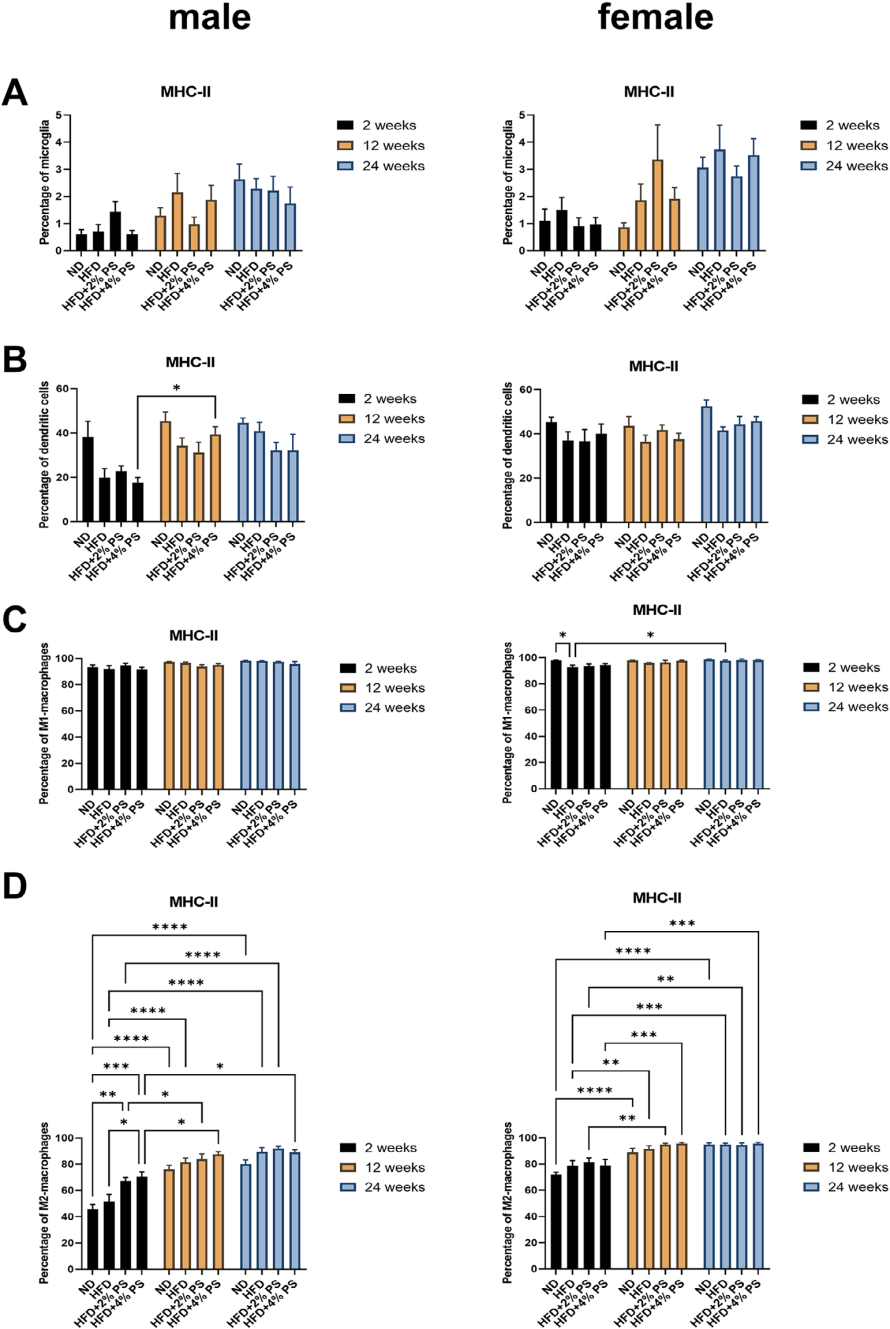
MHC‐II in brain and pancreas. MHC‐II expression from (**A**) microglia Panel 1, (**B**) dendritic cells in pancreas, (**C**) M1‐macrophages in pancreas, (**D**) M2‐macrophages in pancreas after ND, HFD, HFD+2% PS, HFD+4% PS after 2, 12, and 24 weeks of diet in male and female mice (two‐way ANOVA; SEM; *p* value ≤ 0.05).

**FIGURE 5:**
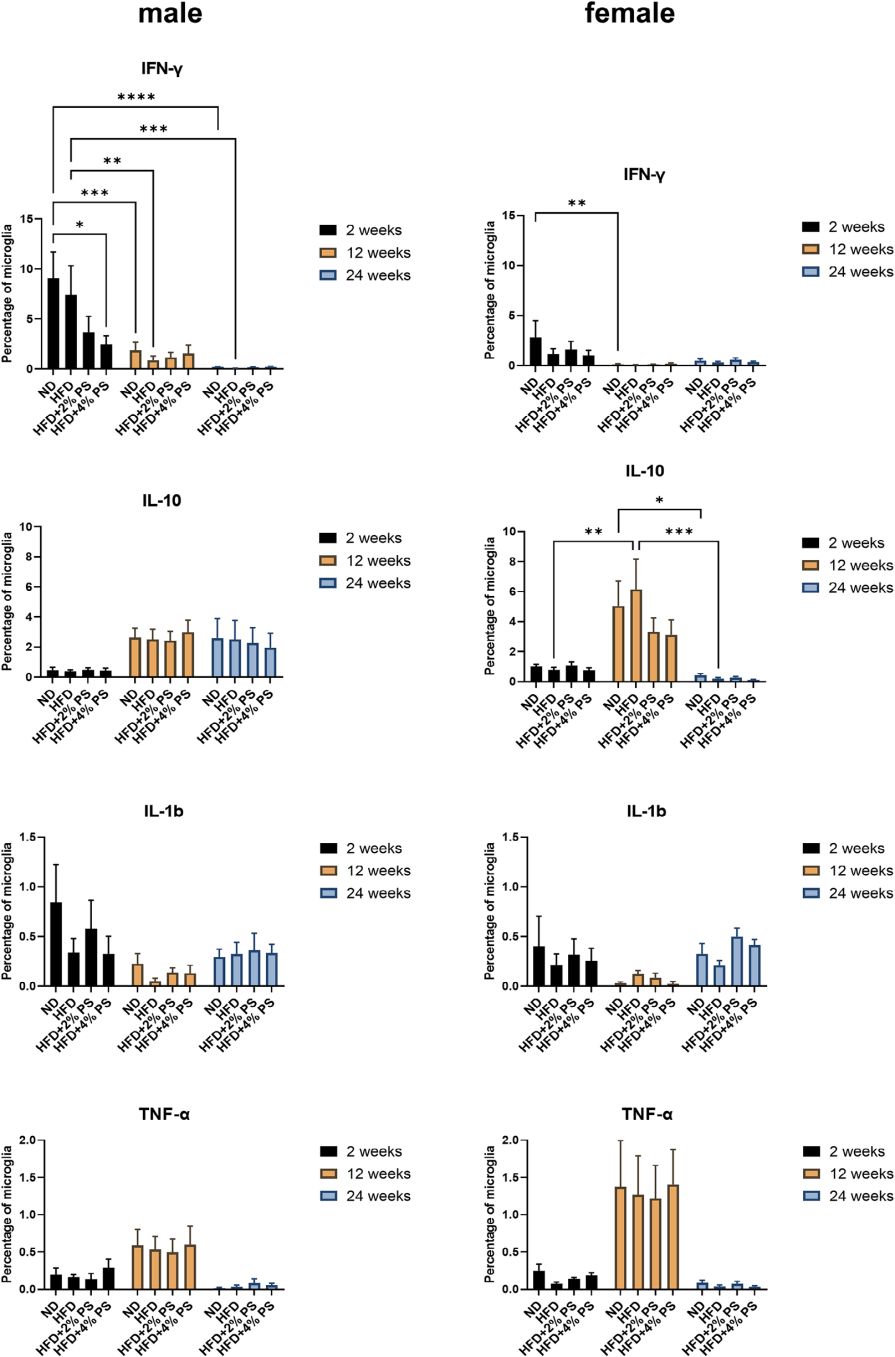
Parenchymal microglia. Expression of pro‐ (IFN‐γ, IL‐1b, TNF‐α) and anti‐inflammatory (IL‐10) cytokines from microglia (Panel 2) after ND, HFD, HFD+2% PS, HFD+4% PS after 2, 12, and 24 weeks of diet in male and female mice (two‐way ANOVA; SEM; *p* value ≤ 0.05).

**FIGURE 6:**
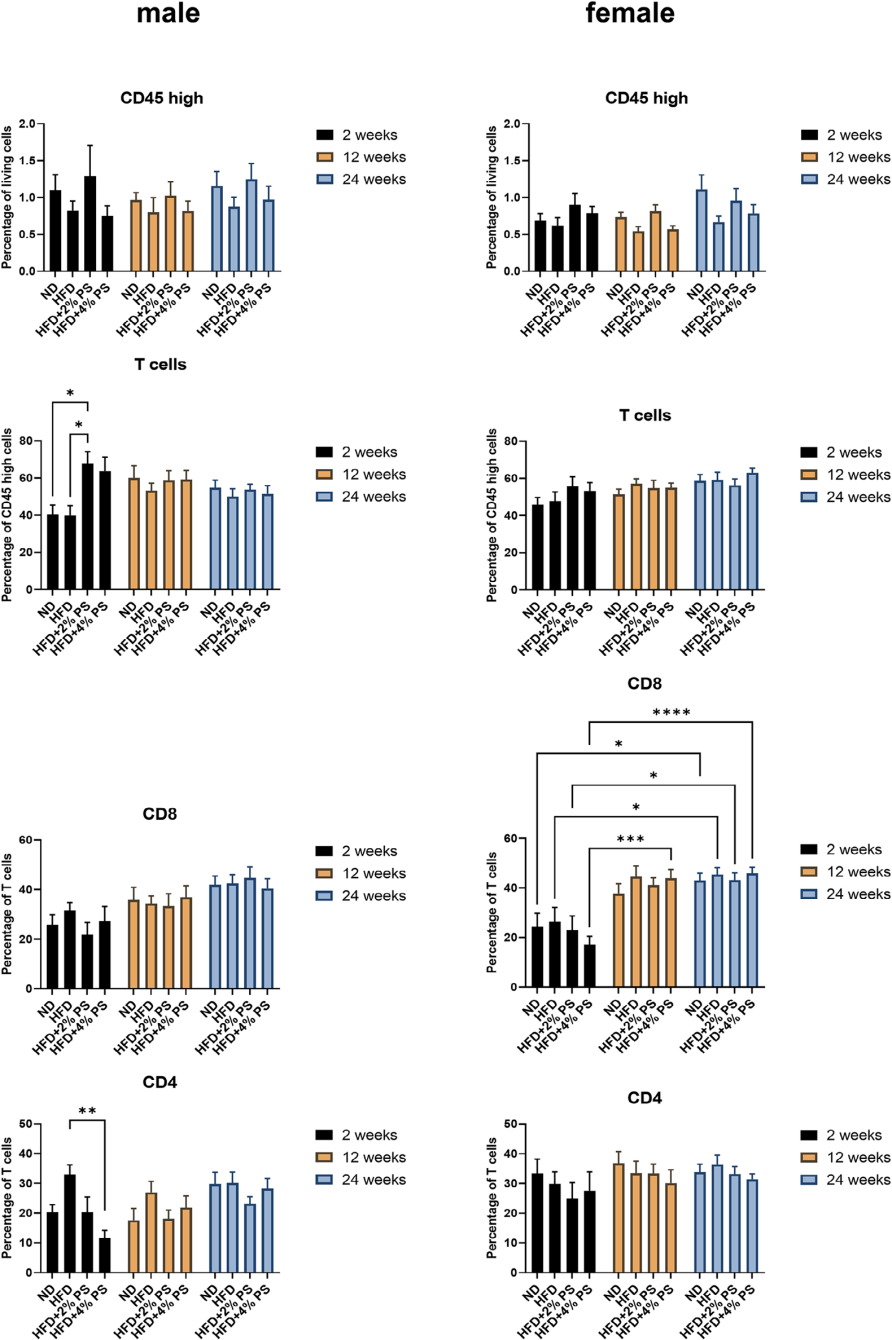
Brain lymphocytes. CD45 high cells, T cells, and expression of CD4‐ and CD8‐positive T cells in brains (Panel 3) after ND, HFD, HFD+2% PS, HFD+4% PS after 2, 12, and 24 weeks of diet in male and female mice (two‐way ANOVA; SEM; *p*‐value ≤ 0.05).

**FIGURE 7:**
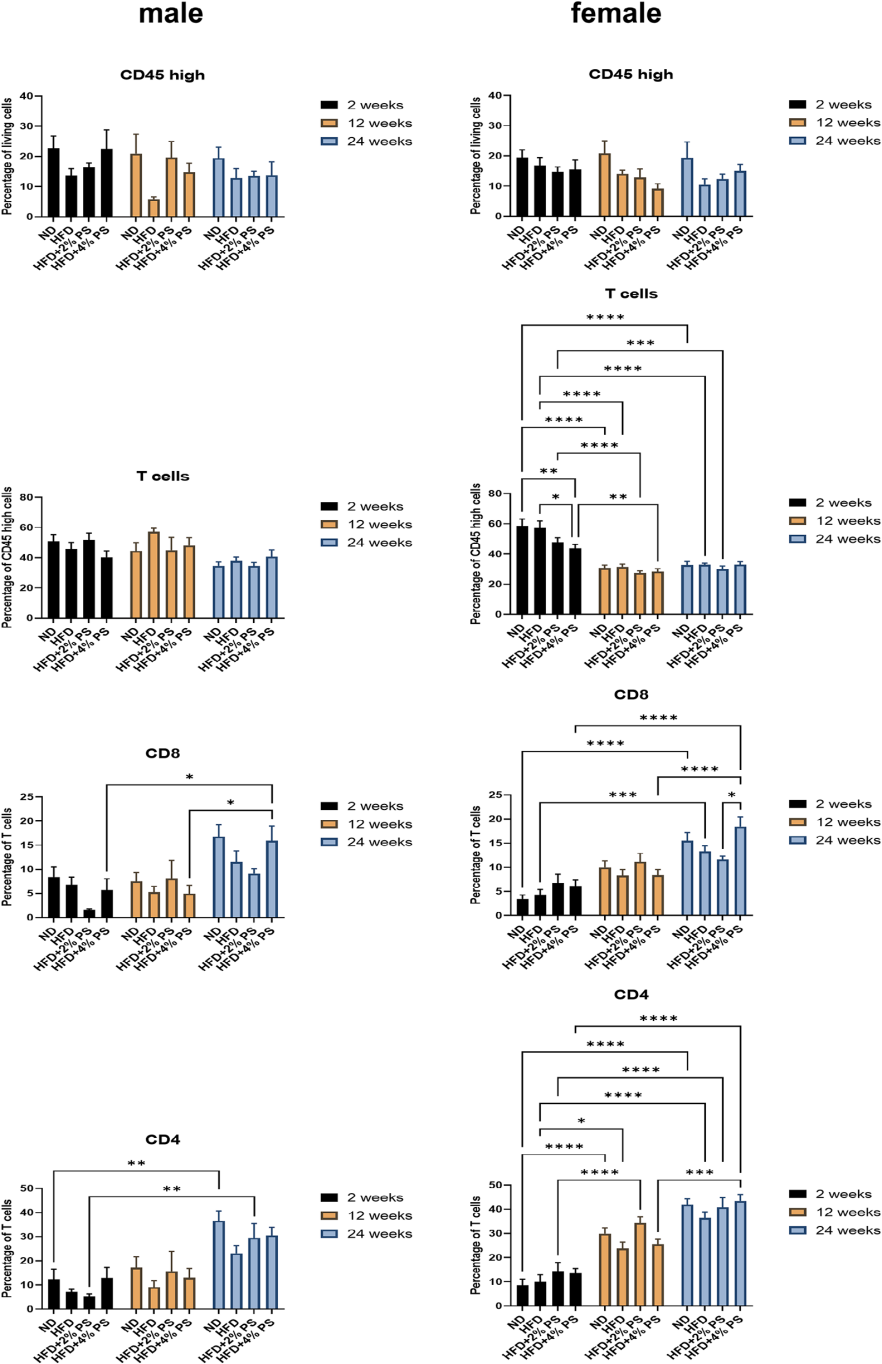
Pancreas lymphocytes. CD45 high cells, T cells, and expression of CD4‐ and CD8‐positive T cells in pancreas after ND, HFD, HFD+2% PS, HFD+4% PS after 2, 12, and 24 weeks of diet in male and female mice (two‐way ANOVA; SEM; *p*‐value ≤ 0.05).

First, striking sex differences were observed, for example, in the numbers of IFN‐γ, IL‐10‐positive microglial cells, and CD4‐positive brain lymphocytes (Figure 3). Therefore, the male and female mice data were analyzed and presented separately. In order to test the impact of HFD versus HFD with PS, we used FC analysis of pancreatic mononuclear cells (Figures 4 and 7) and parenchymal microglia (Figures 4, 5, 6).

The comparison of microglia cells (CD45^int^, CD11b^+^, P2RY12^+^/Gr‐1^‐^) between male and female mice after 2, 12, and 24 weeks on the different diets revealed no significant diet‐induced upregulation of MHC‐II expressing cells (Figure 4A). However, there was an emerging trend toward increased MHC‐II expression over time across all diets (Figure 4).

In dendritic cells (CD45^+^, CD11b^+^, F4/80^−^, CD11c^+^/MHC‐II^+^) from the pancreas (Figure 4B), MHC‐II expression increased from Week 2 to Week 12 in male mice. This increase was significant in the HFD+4% PS group. No significant changes were observed in female mice.

M1‐macrophages (CD45^+^, CD11b^+^, F4/80^+^, CD11c^+^/CD206^−^) of pancreatic leucocytes (Figure 4C) showed no significant regulation of MHC‐II in male mice. In contrast, female mice exhibited a significant decrease in MHC‐II after 2 weeks. Significantly less MHC‐II positive cells were detected in HFD compared to ND. From 2 to 24 weeks, MHC‐II was significantly upregulated in the HFD group.

In M2‐macrophages (CD45^+^, CD11b^+^, F4/80^+^, CD11c^−^/CD206^+^) of pancreatic leucocytes (Figure 4D), male mice exhibited a significant MHC‐II upregulation over time across all diets:

- ND: 2 versus 12, and 2 versus 24
- HFD: 2 versus 12, and 2 versus 24
- HFD+2% PS: 2 versus 12, and 2 versus 24
- HFD+4% PS: 2 versus 12, and 2 versus 24.

Furthermore, after 2 weeks, MHC‐II expression was significantly lower for ND compared to HFD+2% PS and HFD+4% PS. HFD also showed lower expression compared to HFD+4% PS.

In female mice, M2 macrophages exhibited a similar significant increase in MHC‐II expression over time across all diets:

- ND: 2 versus 12, and 2 versus 24
- HFD: 2 versus 12, and 2 versus 24
- HFD+2% PS: 2 versus 12, and 2 versus 24
- HFD+4% PS: 2 versus 12, and 2 versus 24.

However, there were no significant differences between the diets (Figure 4D).

All gating strategies can be found in the Supporting Information S2.

In parenchymal microglia of male mice, IFN‐γ expression was significantly upregulated after 2 weeks of diet compared to 12 and 24 weeks for ND and HFD (Figure 5). The expression of IFN‐γ was also significantly higher for ND than for HFD+4% PS after 2 weeks. In female mice, IFN‐γ expression likewise tended to be higher after 2 weeks on ND compared to the other diets. In addition, a significant downregulation was evident from 2 to 12 weeks for ND.

For IL‐10, a trend toward upregulation was observed in male mice after 12 weeks across all four diets compared to 2 weeks. In female mice, IL‐10 upregulation was determined from 2 to 12 weeks, which was significant for HFD. However, a subsequent downregulation occurred between 12 and 24 weeks which was significant for ND and HFD.

IL‐1b expression showed a downward trend from 2 to 12 weeks in both sexes, followed by an upward trend from 12 to 24 weeks. However, none of these changes reached statistical significance.

TNF‐α levels showed a tendency to increase after 12 weeks in both male and female mice across all four diets, followed by a decrease after 24 weeks (Figure 5).

In CD45^high^ brain cells, no significant regulation was detected in either male or female mice (Figure 6). Moreover, T cell levels (CD45^high^, CD3^+^/CD11b^−^) showed no significant changes in female mice across time or diet. However, in male mice, significantly more T cells were detected after 2 weeks on HFD+2% PS compared to ND and HFD.

No regulation of cytotoxic T cells (CD45^high^, CD3^+^/CD11b^−^, CD8^+^) was detected in male mice. In contrast, female mice showed a significant upregulation over time for all four diets:

- ND: 2 versus 24
- HFD: 2 versus 24
- HFD+2% PS: 2 versus 24
- HFD+4% PS: 2 versus 12, and 2 versus 24.

Nevertheless, no significant differences were observed between the different diets.

For T‐helper cells (CD45^high^, CD3^+^/CD11b^−^, CD4^+^), no significant regulation became evident in female mice. In male mice, the highest levels of T‐helper cells were observed after 2 weeks on HFD, with significantly more compared to HFD+4% PS (Figure 6).

In CD45^high^ pancreatic cells, no significant regulation was evident in male and female mice (Figure 7). Unlike the brain, no regulation of T cell numbers was observed in male mice. In female mice, however, T cells were significantly downregulated over time:

- ND: 2 versus 12, and 2 versus 24
- HFD: 2 versus 12, and 2 versus 24
- HFD+2% PS: 2 versus 12, and 2 versus 24
- HFD+4% PS: 2 versus 12.

Additionally, after 2 weeks, significantly more T cells were detected in the ND and HFD groups compared to HFD+4% PS.

Cytotoxic T cells (CD45^+^, CD3^+^, CD8^+^) were significantly upregulated in male mice after 24 weeks on HFD+4% PS, compared to 2 and 12 weeks. A similar trend was observed for the other three diets, with higher levels of cytotoxic T cells after 24 weeks, though without statistical significance. In female mice, a similar expression was evident. Specifically, significant increases of cytotoxic T cells were observed after 24 weeks of several diets:

- ND: 2 versus 24
- HFD: 2 versus 24
- HFD+4% PS: 2 versus 24, and 12 versus 24.

Moreover, after 24 weeks of diet, HFD+4% PS was associated with significantly more cytotoxic T cells than HFD+2% PS.

Unlike the brain, a time‐dependent regulation of T‐helper cells (CD45^+^, CD3^+^, CD4^+^) was observed in the pancreas. In male mice, a significant upregulation was evident after 24 weeks in the ND and HFD+2% PS groups, relative to 2 weeks. In female mice, upregulation occurred earlier. Significant increases were detected across all diets between 2 and 12 weeks and sustained through Week 24 (Figure 7):

- ND: 2 versus 12, and 2 versus 24
- HFD: 2 versus 12, and 2 versus 24
- HFD+2% PS: 2 versus 12, and 2 versus 24
- HFD+4% PS: 2 versus 24, and 12 versus 24.

In summary, we observed a time‐dependent regulation but not between the different diets. In both brain and pancreatic immune cells, MHC‐II expression increased over time across all diets.

In the brain, cytokine expression patterns also varied with time. IFN‐γ levels were downregulated after 2 weeks in male and female mice. IL‐1b showed a transient downregulation from 2 to 12 weeks, followed by an upregulation from 12 to 24 weeks. TNF‐α levels were upregulated after 12 weeks and downregulated again by 24 weeks. IL‐10 was upregulated after 12 weeks in male mice, while in female mice, it was downregulated again between 12 and 24 weeks. However, no significant differences in the expression of pro‐ or anti‐inflammatory cytokines could be detected that would depend on diets.

### 3.3 Fluorescence Labeling

In order to visualize potential microglial activation depending on diet, we performed immune fluorescence microscopy using IBA1 and TREM2. IBA1 is a microglia marker that is upregulated in activated microglia, but its function is still unclear. In previous studies, we have already shown that long‐term HFD leads to increased microglial activation in the hypothalamus, as indicated by elevated IBA1 fluorescence intensity and percentage of stained area [35]. In addition, we investigated the expression of TREM2, which is a marker of chronic inflammation, by triggering the production of constitutive inflammatory cytokines. TREM2 has been associated with neurodegenerative disorders, including AD. Especially, risk variants of TREM2 are loss of function mutations that contribute to early‐onset AD [42, 43, 44].

**FIGURE 8:**
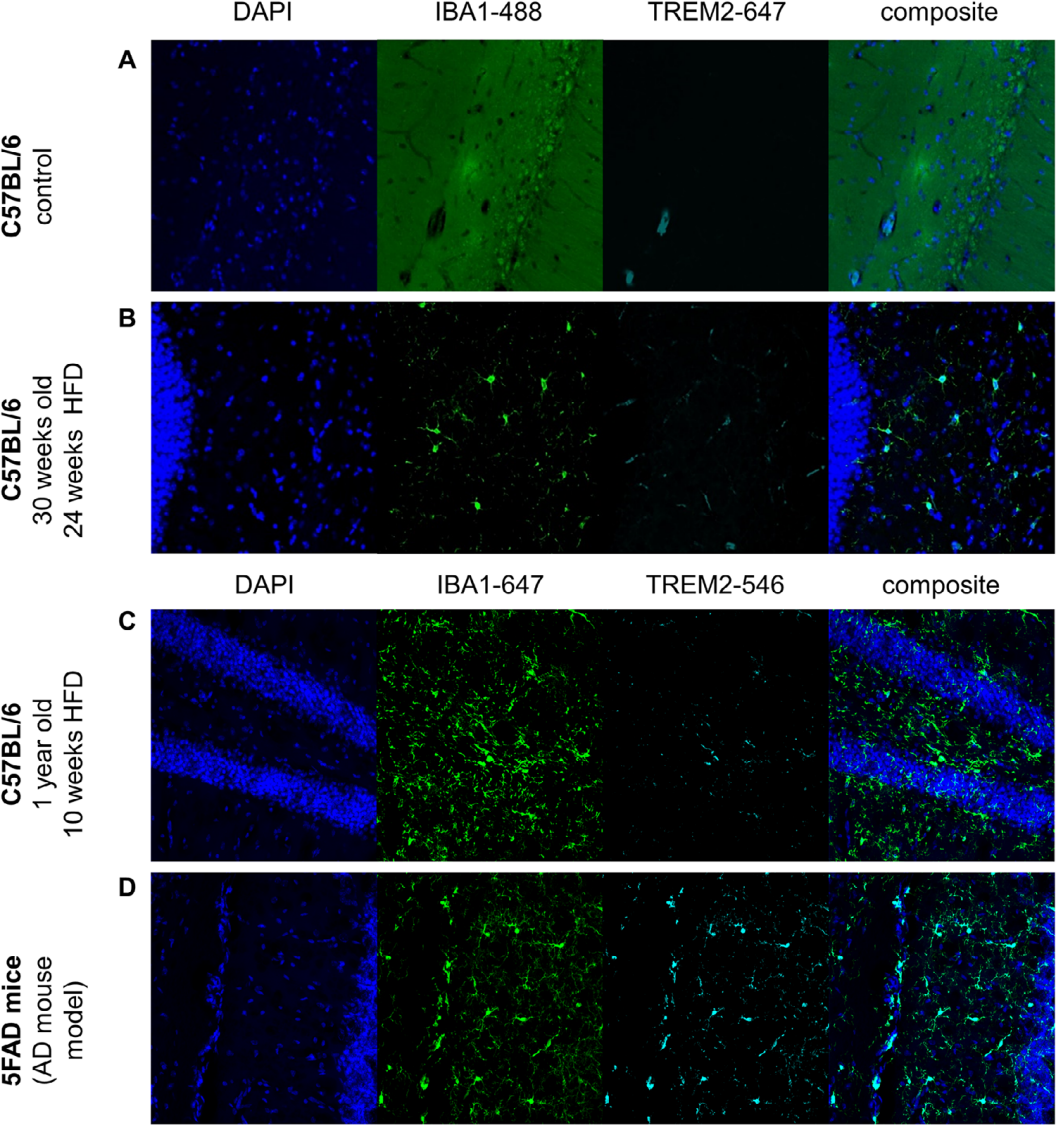
TREM2 staining. Immune fluorescence staining of brain slices (50 µm) of (**A**) control (**B**) 30 weeks old C57BL/6 mouse after 24 weeks of HFD; anti‐IBA1‐488 (green), anti‐Trem2‐647 (dark red), (**C**) 1‐year‐old C57BL/6 mouse after 10 weeks of HFD; anti‐IBA1‐647 (dark red), anti‐Trem2‐546 (red), (**D**) 5FAD mouse (Alzheimer`s mouse model); anti‐IBA1‐647 (dark red), anti‐Trem2‐546 (red); 40× magnification.

Fluorescence labeling of IBA1 (green), TREM2 (cyan), and DAPI (blue) after 24 weeks of HFD (Figure 8B) exhibited no TREM2‐positive cells. As we expected but did not find upregulation of TREM2 upon HFD, we used additional models of HFD treatment and microglial activation. Even in 1‐year‐old mice after 10 weeks of HFD (Figure 8C), no TREM2‐positive cells were detectable. Consequently, no age‐dependent expression of TREM2 was observed. Furthermore, the positive control for TREM2‐antibody was an AD mouse model (5FAD mice) (Figure 8D) that displayed clear co‐localization of IBA1^+^ and TREM2^+^ cells.

## 4 Discussion

In this study, we investigated the effects of ND, HFD, HFD+2% PS, and HFD+4% PS in female and male C57BL/6J mice on indicators of neuroinflammation using FC after different feeding periods (2, 12, and 24 weeks) and immunofluorescence stainings of microglia.

First, we confirmed that the addition of PS to HFD causes significantly less gain of weight than observed upon feeding HFD alone (Figure 1) [45, 46]. PS have been shown to reduce plasma and hepatic triglycerides and to modulate the expression of lipid regulatory genes and de novo lipogenesis in C57BL/6J mice [45]. Therefore, PS consumption could be beneficial for patients with overweight and hypercholesterolemia [47]. In fact, we have shown elsewhere that the concentration of PS in the human brain is inversely associated with body mass index (BMI) [32].

It has been previously published that HFD causes neuroinflammation [8, 9, 18, 48, 49]. Therefore, we performed FC to measure the expression of pro‐ (IFN‐γ, IL‐1β, TNF‐α) and anti‐inflammatory (IL‐10) cytokines in microglia. Interestingly, we found age‐ and sex‐dependent differences, while the diet (ND, HFD, HFD+PS) had no significant impact (Figures 3 and 5). This is in line with our previous observation that HFD has no effect on the learning, memory, reflexes, and emotional state of mice [35]. This suggests that HFD does not always result in inflammation as repeatedly reported [8, 9, 18, 48, 49] and that additional stimuli might be required. These additional stimuli could be different mouse models [50], different diet compositions [51], a later start of the experiment (older mice) [52], or an extended feeding period [53].

Although PS are widely recognized for their antioxidant [54, 55] and anti‐inflammatory properties [56, 57, 58], our findings suggest that their impact on immune regulation under physiological conditions may be limited within the brain and pancreas. This is consistent with a previous study where no substantial differences were observed in low‐ and non‐fat diets supplemented with PS [59]. While PS have demonstrated the capacity to modulate inflammation, particularly under pre‐existing inflammatory conditions [56, 57, 58], their ability to exert broad immunoregulatory or immunosuppressive effects in a non‐inflammatory setting appears to be minimal. The modest influence of PS on immune responses may stem from its mechanism of action. PS are known to attenuate oxidative stress by reducing reactive oxygen species (ROS) and enhancing antioxidant capacity, which in turn can mitigate inflammation [55, 60]. However, these effects are more pronounced in the context of active inflammation or oxidative imbalance, rather than during homeostatic immune function. PS act more as supportive agents in various ways by reducing LDL cholesterol [61], potentially reducing cancer risk [62], providing anti‐inflammatory effects [56, 57, 58], rather than being primary regulators. Therefore, while they may contribute to immune resilience during stress or inflammation, their role in modulating baseline immune function appears limited. The HFD fed in our study did not provide a sufficiently pronounced inflammatory or immunological challenge to fully reveal potential immunoregulatory effects of PS supplementation. This limitation emphasizes the need for future studies to assess the immunological impact of PS under conditions of immune activation or dysregulation to better delineate the contexts in which their bioactivity becomes functionally relevant.

However, we observed an age‐dependent increase in MHC‐II expression after all four diets (Figure 4). It has been previously published that microglial MHC‐II expression is increased in normal aging in humans [63], monkeys [64], and rats [65]. In addition, we observed a regulation of IFN‐γ that was significantly upregulated after 2 weeks of diet compared to 12 and 24 weeks for ND and HFD in male mice and for ND in female mice (Figure 5). IFN‐γ is described as a “booster” of microglial activation, which subsequently acts in a pro‐inflammatory and neurotoxic manner [66]. This leads to microgliosis and enhanced synapse elimination [66]. In another study, microglia cells have been identified as metabolic sensors of nutritional composition [67]. Acute HFD has been found to elicit a homeostatic response that supports cognitive function rather than being neurotoxic [67]. Our findings suggest that microglia become activated by increased IFN‐γ levels after 2 weeks of diet but are downregulated again after 12 weeks and upregulate anti‐inflammatory cytokines (IL‐10) to protect homeostasis. We have no explanation for observing the highest amount of IFN‐γ after 2 weeks of ND. However, HFD supplemented with PS showed anti‐inflammatory effects by tending to downregulate IFN‐γ after 2 weeks (Figure 5).

For IL‐10, a trending upregulation was observed in male mice after 12 weeks for all four diets compared to 2 weeks. In female mice, upregulation was determined from 2 to 12 weeks, which was significant for HFD, and a downregulation again from 12 to 24 weeks, which was significant for ND and HFD (Figure 5). A significant upregulation of IL‐10 mRNA expression has been published in hypothalamic tissue after 8 weeks of HFD feeding in C57BL/6J mice aged 100–120 days [68]. It has been suggested that chronic stimulation causes an anti‐inflammatory response upon an initial inflammatory response [69].

We observed a tendency toward a higher number of T‐helper cells in the brain of male mice after 2 weeks of HFD, which was significantly higher compared to HFD+4% PS (Figure 6). A similar picture emerged for IFN‐γ, which was upregulated by microglia of male mice after 2 weeks of HFD and downregulated after 2 weeks of HFD+4% PS (Figure 5). HFD supplemented with PS showed anti‐inflammatory effects by a trending downregulation of T‐helper cell numbers as described above. Furthermore, T‐helper cell numbers in the brain showed striking sex differences that were significant for ND after 12 weeks of diet (Figure 3). Our findings suggest that male and female mice exhibit distinct immune responses to PS treatment, underscoring the importance of sex as a biological variable in immunological research. This observation aligns with existing literature demonstrating sexual dimorphism in the immune system [70, 71, 72, 73], which manifests in differential susceptibility to infections [74, 75], autoimmune diseases [76, 77], cancer prevalence [78, 79], and variability in responses to vaccines [80, 81]. The sex‐specific effects observed in our study may be attributed to both genetic and hormonal factors. Sex chromosomes, particularly the X chromosome, encode several immune‐related genes that can differentially affect immune cell development and function [82, 83]. In addition, sex hormones such as estrogens, progesterone, and androgens modulate numerous aspects of innate and adaptive immunity, including cytokine production, antigen presentation, and lymphocyte activity [84, 85, 86, 87]. In the pharmaceutical industry, women have only been systematically included in studies in the European Union (EU) since 2005, when Directive 2001/20/EC on the harmonization of clinical trials came into force [88]. Historically, medical studies were performed with male participants, as they do not experience hormonal fluctuations, menopause, and cannot become pregnant [89]. Accordingly, the research data collected from men was then generalized to women [90, 91].

Since PS are bioactive compounds capable of influencing immune pathways [56, 57, 58], it is conceivable that their effects are modulated by the hormonal milieu, leading to divergent outcomes in males and females. These findings emphasize the necessity of incorporating sex as a factor in experimental design and data interpretation when assessing the immunological impacts of dietary or pharmacological interventions.

It has been published that women have higher levels of IL‐10 produced by CD4^+^ T‐helper cells and regulatory T cells [92]. Furthermore, females exhibit a higher number of CD4^+^ T cells than age‐matched males [93, 94, 95]. These emerging sex‐specific differences seem to occur from a combination of sex hormone‐derived influences and cell‐intrinsic influences as described above. For example, these may be due to the estrogen 17‐beta‐estradiol [96] and differential gene expression through X‐linked epigenetic regulation [97].

In addition, pancreatic lymphocytes showed an age‐dependent regulation. Both cytotoxic T‐cell numbers and T‐helper cell numbers were upregulated in the course of aging in all four diets (Figure 7). We observed greater diet‐related differences in the number of pancreatic lymphocytes (Figure 7) than in brain lymphocytes (Figure 6). This suggests that inflammation was already present in the periphery. It has been previously published that mice fed an HFD develop obesity‐induced inflammation with local proliferation of macrophages in adipose tissue [98, 99]. In the brain, an anti‐inflammatory milieu prevails [100, 101], which may underlie our observation.

In our study, the time‐dependent regulation of cytokines appeared to be more pronounced than the influence of dietary composition. Nevertheless, we do not conclude from our observations that time, in the form of the feeding period or aging, generally has a greater influence on immune activation than the composition of the diet. It has already been described several times that nutrition plays an essential role in the regulation of the immune system and that malnutrition influences life expectancy because it impairs the effectiveness of the immune response [102, 103, 104, 105]. Therefore, rather than viewing nutrition and duration of the diet or aging as isolated variables, their interaction must be considered in combination in terms of immune regulation.

The results of our study suggest that HFD alone does not trigger neuroinflammation in the absence of prolonged exposure or additional risk factors. Transferring these findings to humans, factors such as genetic predisposition, pre‐existing conditions, and the influence of years of malnutrition must not be disregarded. Encouragingly, our results suggest that temporary exposure to HFD does not induce detectable neuroinflammation in healthy mice without pre‐existing diseases and genetic predispositions. Nonetheless, obesity shows well‐documented effects on immune function [102, 103]. It alters the primary lymphoid organs, such as bone marrow and thymus, contributing to age‐related regression, and impairment of lymphopoiesis and immune function [102, 103]. The mucous membranes, as part of the secondary lymphoid organs, are also affected by increased permeability [106, 107]. Furthermore, micronutrient deficiencies can negatively affect primary and secondary lymphoid organs. Deficiencies of zinc, iron, selenium, and B vitamins are associated with changes in bone marrow and thymus [102, 108, 109], whereas vitamins A, D, and C are essential for maintaining mucosal integrity, and their deficiency is associated with mucosal dysfunction [102, 108]. A next step in understanding immune regulation is to investigate molecular regulators such as TREM2, which may mediate the brain's response to metabolic and inflammatory signals.

TREM2 is upregulated on microglia under various neurodegenerative and inflammatory conditions [110]. Loss of function mutations are associated with the development of AD [111, 112]. In APP‐overexpressing mice, *Trem2* is the highest‐regulated gene at amyloid plaques [42]. Increased expression of TREM2 on microglia is coupled with increased phagocytosis, which promotes the activation state of microglia, acting protective by clearing toxic products (such as amyloid protein) [42, 111]. Accordingly, reduced TREM2 activity results in the brain's inability to clear these detrimental compounds [112]. On the other hand, TREM2 suppresses pro‐inflammatory cytokine production and secretion [42, 111]. Therefore, the upregulation of TREM2 has been published as a compensatory response of the brain to alleviate neurological damage due to chronic inflammation. Lack of TREM2 lock microglia in a homeostatic state [113].

We expected upregulation of TREM2 upon HFD based on other studies that observed increased TREM2 levels in the HFD mice brains [114, 115, 116]. In addition, we have published increased microglial activation after long periods of HFD in the hypothalamus by IBA1 stainings [35]. Likewise, a study by Zemer et al. (manuscript under review) showed that 8 weeks HFD induced activation of microglia in the hypothalamus in adult and healthy middle‐aged mice, as observed by IHC. However, in our study, we did not detect any TREM2 signals after HFD, while positive controls exhibited vivid staining (Figure 8). This could be explained by the previous observations that additional stimuli to HFD might be required to induce neuroinflammation. The observations by Zemer et al. support our hypothesis that aged mice may be an additional stimulus favoring inflammation in the brain. Another explanation would be that microglial activation may be region‐specific and can only be observed by high‐resolution analysis of specific areas, such as the nucleus arcuatus.

We have previously shown that PS inversely correlate with inflammatory markers in the brain [32]. In addition, reduced in vitro microglial activation has been observed after the addition of PS [32]. However, several limitations must be acknowledged. In the present study, the chosen HFD model did not elicit a sufficiently inflammatory phenotype to allow a conclusive evaluation of PS effects on immune regulation or neuroinflammatory processes. Therefore, further experiments are required to investigate whether adding physiological PS to an obesity‐associated HFD positively affects neuroinflammation and the underlying signaling pathways. Another approach could be genetically modified mouse models, such as ob^−/−^ or APP transgenic mice, that mimic aspects of obesity and AD. In these mouse models, neuroinflammation is more pronounced, and systemic immune dysregulation is evident. In addition, aged mice could provide insights into age‐related immune senescence. Chronic low‐grade inflammation potentially enhances the sensitivity to PS intervention. Furthermore, longer feeding periods could result in the manifestation of cumulative effects of PS supplementation.

In conclusion, we observed a time‐dependent and a sex‐specific regulation. Supplementation of PS to HFD resulted in a reduction in weight gain, indicating potential metabolic benefits. Further studies with different approaches mentioned above will improve our understanding of the therapeutic potential of PS.

## Conflicts of Interest

The authors declare no conflict of interest. The funders had no role in the design of the study; in the collection, analyses, or interpretation of data; in the writing of the manuscript, or in the decision to publish the results.

## Supporting information




**Supporting File 1**: mnfr70168‐sup‐0001‐SuppMatS1.pdf.

**Supporting File 2**: mnfr70168‐sup‐0002‐SuppMatS2.pdf.

## Data Availability

All relevant data are within the manuscript and its Supporting Information files.
