# Supplementary material for: Exploring the Impact of a High‐Fat Diet on Brain Homeostasis: A Comprehensive Analysis of the Absence of Inflammation
Source: Mol Nutr Food Res. 2025 Jul 28;69(20):e70168. doi: 10.1002/mnfr.70168 (PMC12538544; doi:10.1002/mnfr.70168)
Supplement: Supplementary file 1 — Supporting File 1: mnfr70168‐sup‐0001‐SuppMatS1.pdf. [file MNFR-69-e70168-s001.pdf]

S1 – Diet composition

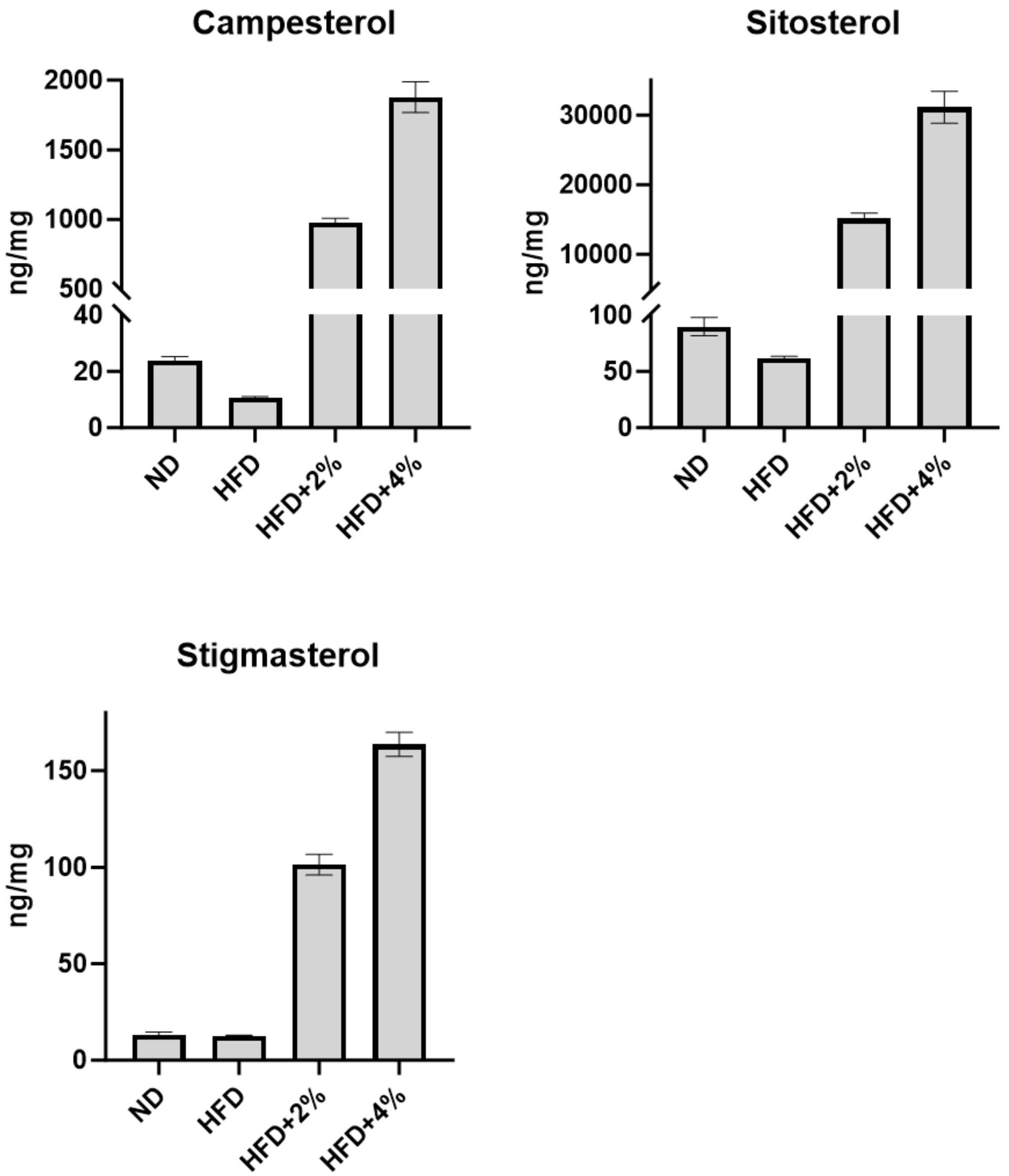

Fig 1: PS-determination by mass spectrometry. Determination of the phytosterols campesterol, sitosterol, and stigmasterol from the food pellets fed to mice over different feeding periods.

ssniff® EF R/M D12331 mod.* / Surwit

Experimental diet with very high fat content (hydrogenated coconut oil) / DIO

Description

The experimental diet is characterized by extremely high amounts of fat with middle-chain, saturated fatty acids (coconut oil); because of its high fat content the feed will quickly induce obesity and and may promote the development of Metabolic Syndrome with diabetes type 2 (NIDDM). The feeding period until first clinical symptoms might be observed depends largely on the rat or mouse strain and the previous dietary history (fat supply).

Crude Nutrients	[%]
Dry matter	97.1
Crude protein (N x 6.25)	21.6
**Crude fat**	**35.7**
Crude fibre	0.1
Crude ash	5.2
N free extracts	34.1
Starch	0.9
**Sugar**	**17.1**
Dextrines	16.5

[1] *ME calculated according to the pig formula, Annex 4 of the German feed regulation*

[2] *ME calculated with the Atwater factors*

Energy	[MJ/kg]
**Gross Energy (GE)**	**25.1**
**Metabolizable Energy (ME)**	

22.6 [1)]

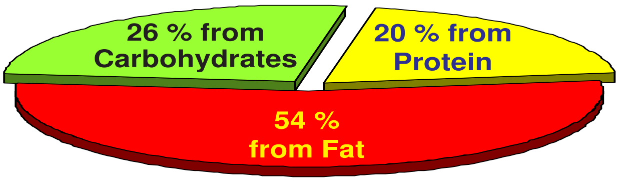

22.8 [2)]

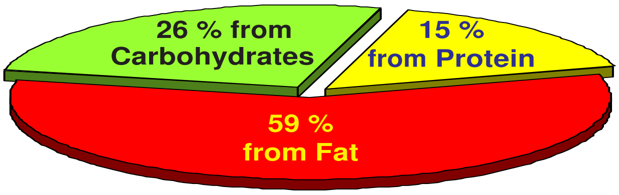

Minerals	[%]
Calcium	0.86
Phosphorus	0.60
Sodium	0.34
Magnesium	0.20
Potassium	0.90

Fatty acids	[%]
C 6:0	0.20
C 8:0	2.53
C 10:0	2.00
C 12:0	14.96
C 14:0	5.75
C 16:0	3.14
C 16:1	0.02
C 17:0	—
C 18:0	1.05
C 18:1	2.86
C 18:2	1.82
C 18:3	0.15
C 20:0	0.01
C 20:1	0.01
C 20:5	—
C 22:6	—
Cholesterol [mg/kg]	—

Amino acids	[%]
Lysine	1.64
Methionine	0.81
Cystine	0.19
Met+Cys	1.00
Threonine	0.89
Tryptophan	0.26
Arginine	0.73
Histidine	0.63
Valine	1.36
Isoleucine	1.04
Leucine	1.96
Phenylalanine	1.07
Phe+Tyr	2.13
Glycine	0.42
Glutamic acid	4.49
Aspartic acid	1.48
Proline	2.29
Alanine	0.65
Serine	1.19

Vitamins	per kg	
Vitamin A	15,000	IU
Vitamin D_3	1,500	IU
Vitamin E	154	mg
Vitamin K (as menadione)	20	mg
Vitamin C	30	mg
Thiamin (B_1)	16	mg
Riboflavin (B_2)	16	mg
Pyridoxine (B_6)	17	mg
Cobalamin (B_{12})	30	µg
Nicotinic acid	46	mg
Pantothenic acid	55	mg
Folic acid	19	mg
Biotin	310	µg
Choline-Chloride	1,050	mg
Inositol	80	mg

Trace elements	per kg	
Iron	151	mg
Manganese	90	mg
Zinc	60	mg
Copper	13	mg
Iodine	1.05	mg
Selenium	0.14	mg
Cobalt	0.14	mg

Feed composition
On request

Main products

E15772-30 Meal
E15772-34 10 mm pellets
(orange colour)

Production and sale
ssniff Spezialdiäten GmbH
Phone:+49-(0)2921-9658-0
Fax: +49-(0)2921-9658-40
E-Mail mail@ssniff.de
www.ssniff.de / www.ssniff.com

* *Research Diets Inc.*

We create chemistry

Vegapure® 867 G E

Chemical names of active ingredient

Plant sterols, phytosterols

PRD-No.

30798810

Articles

50781770	15 kg bag in box

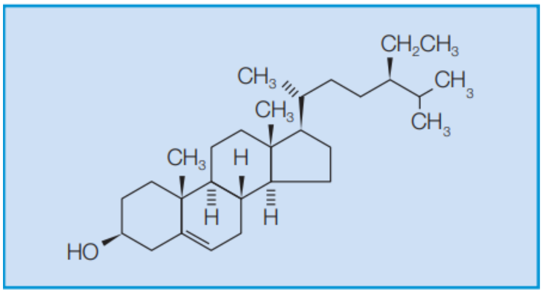

Main active: Beta Sitosterol

Country of origin

USA (micronized in Germany)

Description

Vegapure® 867 G E plant sterols are a finely ground powder of naturally occurring phytosterols derived from pine trees. It is rich in Beta Sitosterol.

Composition

Ingredients in descending order of weight:
Plant sterols.

Solubility

Soluble in organic solvents, poor soluble in vegetable oils and fats; insoluble in water.

Standards

Product is kosher and halal.
Please see document „Quality and Regulatory Product Information“ (QRPI).

Specification

Assay	
Total sterol content	min. 99%

For further information see separate document “Product Specification”.

Regulations

Plant sterols have GRAS (generally recognized as safe) status for use in food applications in the USA. They have also been approved as Novel Food ingredient for several specific functional food applications within the EU as well as in many other countries. However, specific regulations on the product and its ingredients in the respective countries and for its intended use have to be observed. Vegapure® 867 G E is derived from not genetically modified raw materials and is thus not subject to labelling under EU Regulations (EC) 1829/2003 and 1830/2003.

Stability, Storage and Handling

Stored in its unopened original packaging at ambient temperatures (max. 25 °C), the product is stable for at least 48 months.
Store under cool and dry conditions in tightly closed containers. Protect from heat, light and humidity. Powdered material may form dust explosive dust-air mixtures.

Applications

Food and beverage products:
Vegapure® 867 G E is intended for use in functional food prepared through dry mixes such as sterol enriched bread. The product can also be applied in cloudy beverages such as juices via high shear mixing/high pressure homogenization. It is almost neutral in taste and odor.

Dietary supplements:
Vegapure® 867 G E is also intended for use in supplements. Selected applications are hard shell capsules and tablets.

Notes

Vegapure® 867 G E must be handled in accordance with the Material Safety Data Sheet.

This document and any information provided herein does not constitute a legally binding obligation of BASF and has been prepared in good faith and is believed to be accurate as of the date of issuance. Unless expressly agreed otherwise in writing in a supply contract or other written agreement between you and BASF:

a) To the fullest extent not prohibited by the applicable laws, BASF EXPRESSLY DISCLAIMS ALL REPRESENTATIONS, WARRANTIES, CONDITIONS OR GUARANTEES OF ANY KIND, WHETHER EXPRESS OR IMPLIED, WRITTEN OR ORAL, BY FACT OR LAW, INCLUDING ANY IMPLIED WARRANTIES, REPRESENTATIONS OR CONDITIONS OF MERCHANTABILITY, FITNESS FOR A PARTICULAR PURPOSE, SATISFACTORY QUALITY, NON-INFRINGEMENT, AND ANY REPRESENTATIONS, WARRANTIES, CONDITIONS OR GUARANTEES, ARISING FROM STATUTE, COURSE OF DEALING OR USAGE OF TRADE AND BASF HEREBY EXPRESSLY EXCLUDES AND DISCLAIMS ANY LIABILITY RESULTING FROM OR IN CONNECTION WITH THIS DOCUMENT OR ANY INFORMATION PROVIDED HEREIN, including, without limitation, any liability for any direct, consequential, special, or punitive damages relating to or arising therefrom, except in cases of (i) death or personal injury, (ii) BASF´s or its agents and assistants willful misconduct, fraud or fraudulent misrepresentation or (iii) any matter in respect of which it would be unlawful for BASF to exclude or restrict liability under the applicable laws;
b) Any information provided herein can be changed at BASF´s sole discretion anytime and neither this document nor the information provided herein may be relied upon to satisfy any obligations you may have to undertake your own inspections and evaluation;
c) BASF rejects any obligation to, and will not, automatically update this document and any information provided herein, unless required by applicable law; and
d) You are responsible for confirming that you have retrieved the most current version of this document from BASF.
e) This document or any information provided herein must not be used for purposes of pharmaceutical registrations.

If you have any further questions or need additional support, please contact your BASF sales representative.

Product Specification	*FINAL*	Effective from	01.11.2018

Vegapure® 867 G N **PRD-No. 30585308**

® = Registered trademark of BASF group ™ = Trademark of BASF group 136229 **Revision 5**

NOT FOR REGULATORY PURPOSES

Test parameter	Requirement	Test method
**Characters**		
Appearance	white to off-white powder	Visual
Odor	bland	Sensory
**Tests**		
Particle size, D90	≤ 150 µm	QC 001.16
Particle size, D50	20 to 40 µm	QC 001.16
Moisture	≤ 1 %	QC-621b
Heavy metals (as Pb)(*)	≤ 10 ppm	USP 231 Method II
Lead (Pb)(*)	≤ 0.1 mg/kg	ICP-MS
Arsenic (As)(*)	≤ 0.1 mg/kg	ICP-MS
Mercury (Hg)(*)	≤ 0.05 mg/kg	CVAA
Cadmium (Cd)(*)	≤ 1.0 mg/kg	ICP-MS
Antimony(*)	≤ 1.0 mg/kg	ICP-MS
Tin(*)	≤ 50 mg/kg	ICP-MS
Sterol composition:		
Total sterols	≥ 99 %	QC-186A
Brassicasterol	≤ 3.0 %	QC-186A
Campesterol	≤ 15.0 %	QC-186A
Campestanol	≤ 5.0 %	QC-186A
Stigmasterol	≤ 5.0 %	QC-186A
Beta-Sitosterol	67.0 to 80.0 %	QC-186A
Beta-Sitostanol	≤ 15.0 %	QC-186A
other sterols/stanols	≤ 3.0 %	QC-186A
Total aerobic plate count	< 1000 CFU/g	USP <61>
Yeast and mould	< 100 CFU/g	USP <61>
Bacillus cereus	< 10 CFU/g	FDA BAM CH 14
Enterobacteriaceae	< 10 CFU/g	AOAC OMA 2003.01
Salmonella	Negative/25 g	AOAC OMA 2011.03

Product Specification	*FINAL*	Effective from	01.11.2018

Vegapure® 867 G N **PRD-No. 30585308**

® = Registered trademark of BASF group ™ = Trademark of BASF group 136229 **Revision 5**

(*) ensured by quality assurance measures and therefore tested randomly only.

The product meets the requirements of this specification.

This document is valid without signature.

The aforementioned data shall constitute the agreed contractual quality of the product at the time of passing of risk. The data are controlled at regular intervals as part of our quality assurance program. SELLER MAKES NO WARRANTY OF ANY KIND, EITHER EXPRESS OR IMPLIED, BY FACT OR LAW, INCLUDING WARRANTIES OF MERCHANTABILITY OR FITNESS FOR A PARTICULAR PURPOSE. The statements under the heading “Characters” are not to be interpreted in a strict sense and are not requirements.
