# Supplementary material for: Exploring the Impact of a High‐Fat Diet on Brain Homeostasis: A Comprehensive Analysis of the Absence of Inflammation
Source: Mol Nutr Food Res. 2025 Jul 28;69(20):e70168. doi: 10.1002/mnfr.70168 (PMC12538544; doi:10.1002/mnfr.70168)
Supplement: Supplementary file 2 — Supporting File 2: mnfr70168‐sup‐0002‐SuppMatS2.pdf. [file MNFR-69-e70168-s002.pdf]

S2 – Gating strategies and isolation of pancreatic leucocytes

2 | Materials and methods

2.2 | Isolation of pancreatic leucocytes

Animals were killed by CO_2 and subsequently perfused with ice-cold PBS (Gibco, Life Technologies, Darmstadt, Germany) to clear the intravascular compartment from blood cells. The pancreas was removed, transferred into ice-cold HBSS (-$CaCl_2$, -$MgCl_2$, Gibco) + 10 % FBS (Biowest, Nuaillé, France), and rapidly minced within the tube using scissors. Subsequently, the tissue was washed twice at 1,200 rpm at 4 °C for 10 min. Digestion was performed in 1.5 ml HBSS + 10 % FBS with 2 mg/ml Collagenase IV (Worthington Biochemical Cooperation, Lakewood, US) using a thermomixer (Eppendorf, Hamburg, Germany) by 1,400 rpm at 37 °C for 30-45 min depending on the tissue sample. Samples were rapidly shaken vertically by hand in between (every 5 min) to improve digestion. After 20 sec vortexing at low speed, digestion was stopped by adding 150 µl FBS, and cell suspension was filtered through a 70 µm cell strainer (BD Falcon, BD Biosciences, Heidelberg, Germany). Samples were washed twice with HBSS containing 2 % BSA (Carl Roth, Karlsruhe, Germany) at 1,200 rpm at 4 °C for 10 min. Afterward, cells were stained for flow cytometry (see below).

2.3 | Flow cytometry staining – pancreatic leucocytes

Cells were pre-incubated with anti-CD16/32 antibody (1:100, eBioscience, San Diego, CA) for 10 min to minimize unspecific binding of antibodies on Fc-receptors. Next, cells were incubated for 30 min with primary labeled antibodies on ice in the dark: **Panel A:** CD45-Fluor780 (1:100, Thermo Fisher), CD11b-PeCy7 (1:100, Biolegend), CD11c-BV605 (1:100, Biolegend), MHC-II-PB (1:100, Biolegend), F4/80-AF488 (1:100, Thermo Fisher), CD301-AF647 (1:50, BioRad); **Panel B**: CD45-Fluor780 (1:100, Thermo Fisher), CD4-PE (1:100, Biolegend), CD8b-AF647 (1:100, Biolegend), CD3-PECy7 (1:100, Biolegend). Afterward, all samples were labeled with live/dead dye (zombie UV^{TM} Fixable Viability Kit, eBioscience) for 30 min at room temperature. Cells were washed with wash buffer (BD Perm/Wash^{TM} Buffer, eBioscience) and centrifuged for 6 min at 1,100 rpm at 4 °C. Supernatant was discarded and cells were fixed with 100 µl Fixation/Permeabilization solution (eBioscience) for 20 min on ice. Cells were washed with wash buffer and centrifuged for 5 min at 1,200 rpm at 4 °C. Supernatant was discarded and samples of Panel B were rinsed in 200 µl PBS. To stain for intracellular marker in Panel A fixed/permeabilized cells were re-suspended in 100 µl of PBS containing CD206-RPE (1:10, BioRad) and incubated on ice for 30 min in the dark. Cells were washed once again with PBS and centrifuged for 5 min at 1,200 rpm at 4 °C. Supernatant was discarded and samples of panel A were also rinsed in 200 µl PBS. For all flow cytometry stainings gates were set accordingly to blank and isotype controls in each experiment.

2.3.1 | Gating strategies

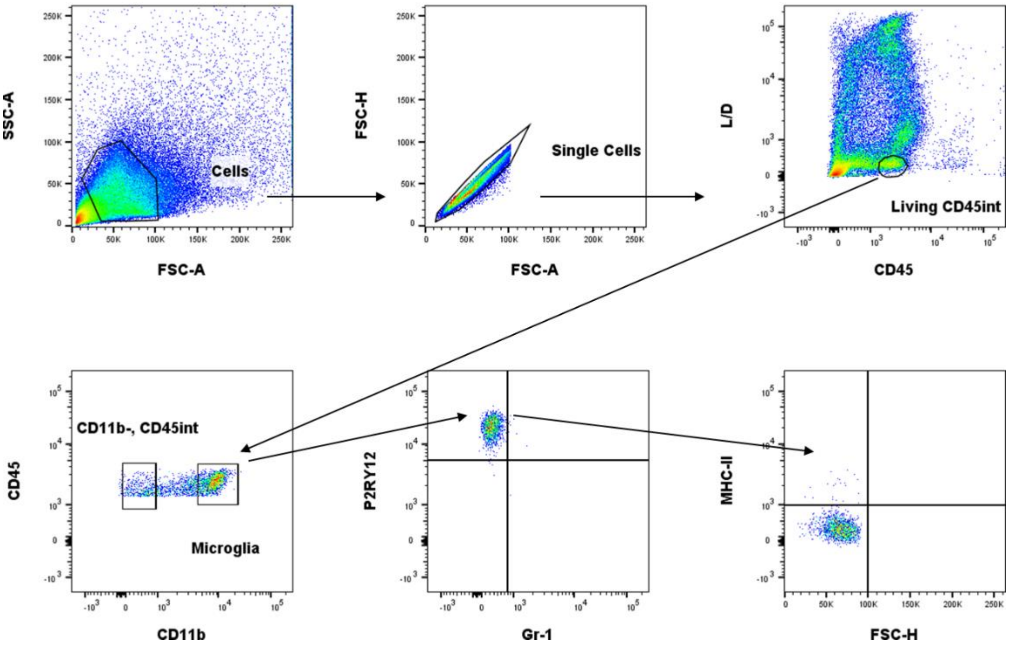

Fig 1: Flow cytometry gating strategy panel 1 (CD45^{int}/CD11b^{+}, P2RY12^{+}/Gr-1^{-}, MHC-II^{+}) of isolated microglia.

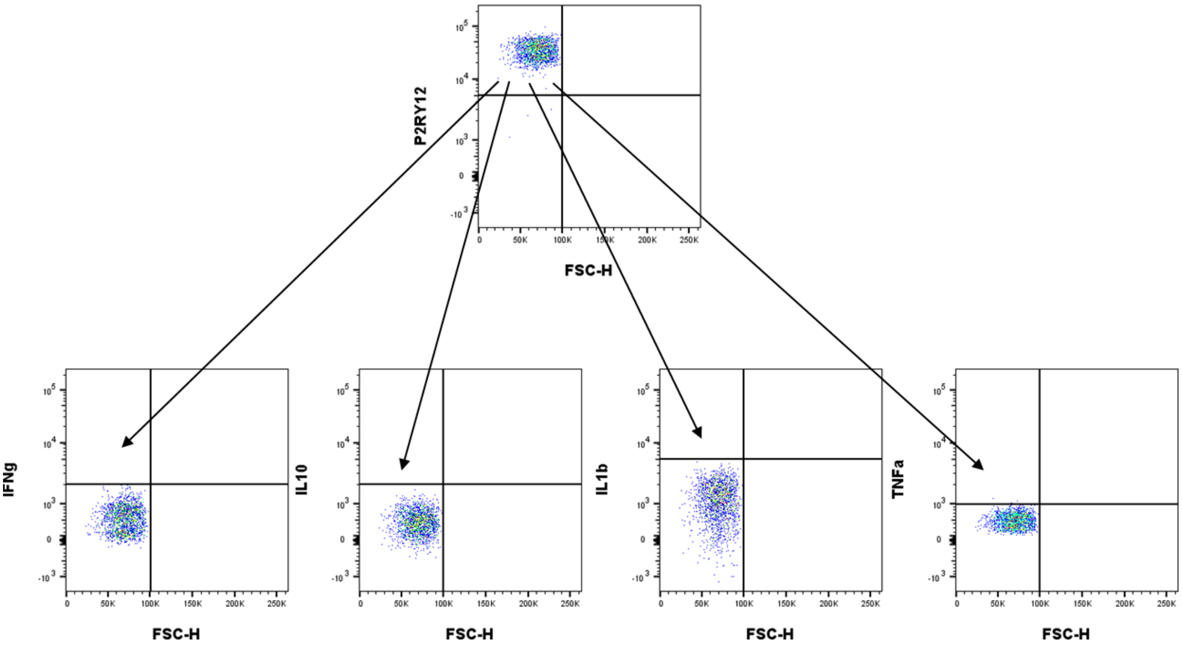

Fig 2: Flow cytometry gating strategy panel 2 (CD45^{int}/CD11b^{+}, P2RY12^{+}, TNF-α^{+}, IL-10^{+}, IL-1β^{+}, INF-γ^{+}) of isolated microglia.

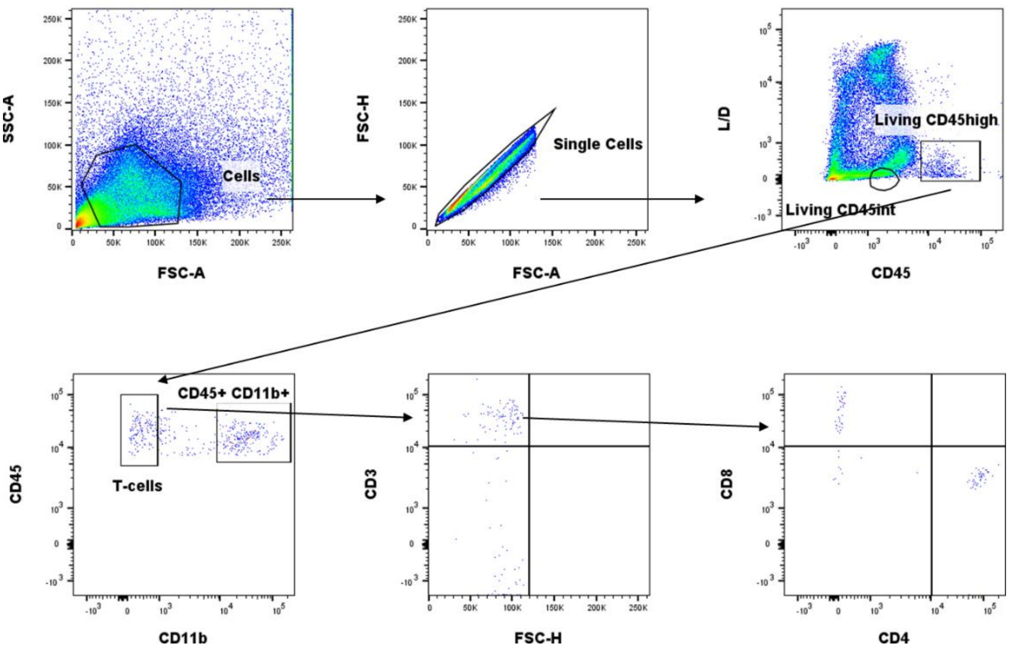

Fig 3: Flow cytometry staining panel 3 (CD45^{high}, CD11b^{-}, CD3^{+}, CD4^{+}, CD8^{+}) of isolated microglia.

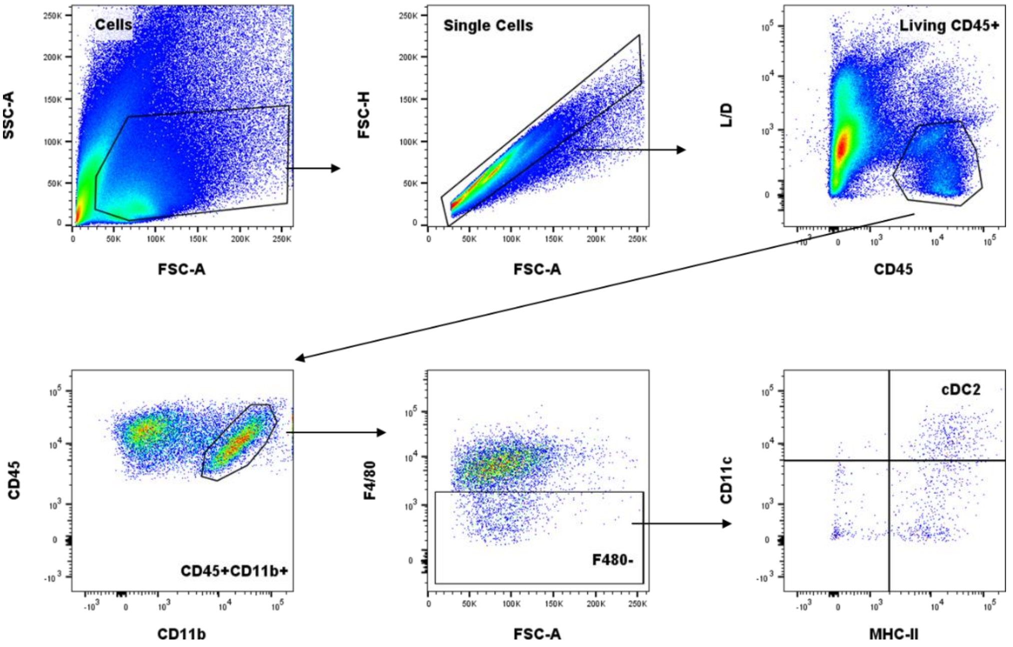

Fig 4: Flow cytometry gating strategy (CD45^{+}, CD11b^{+}, F4/80^{-}, CD11c^{+}/MHC-II^{+}) of isolated pancreatic cells to differentiate dendritic cells.

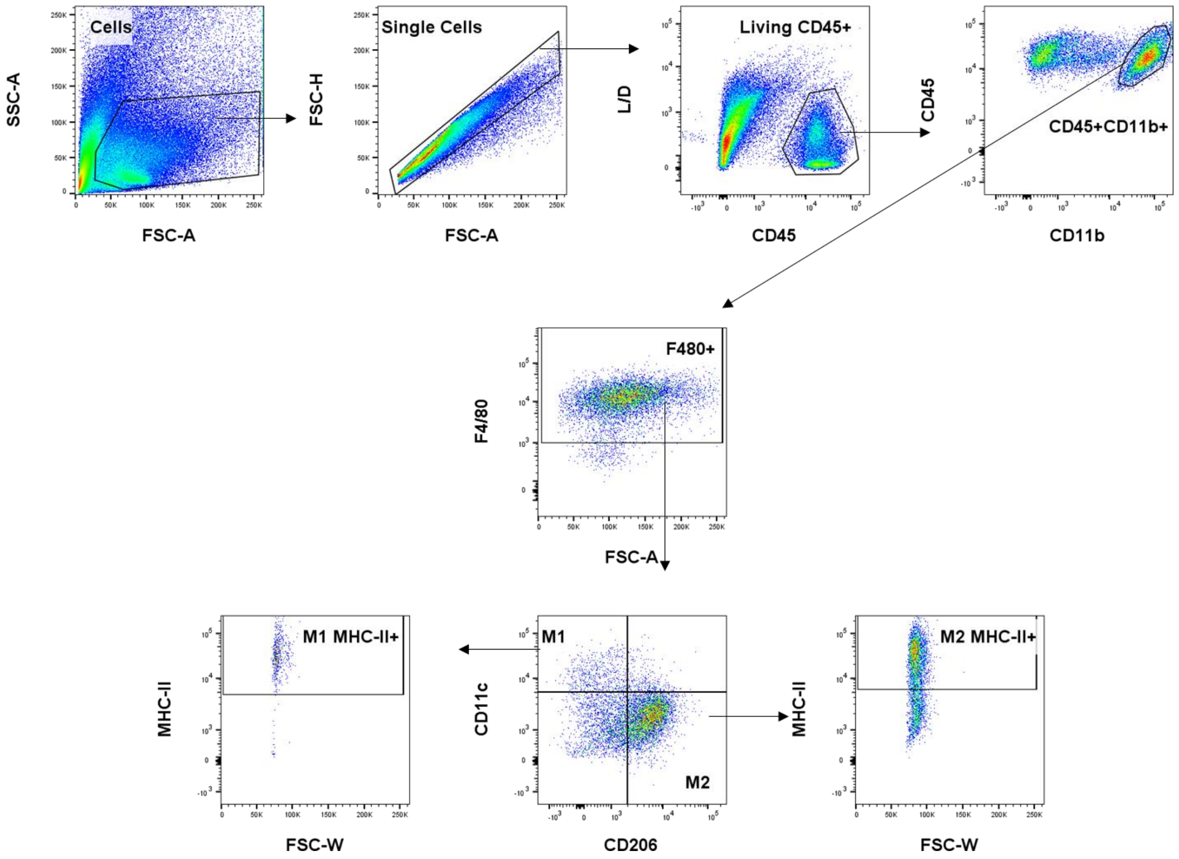

Fig 5: Flow cytometry gating strategy (CD45$^+$, CD11b$^+$, F4/80$^+$, CD11c$^+$/CD206$^-$, CD11c$^-$/CD206$^+$) of isolated pancreatic cells to differentiate M1- and M2-macrophages.

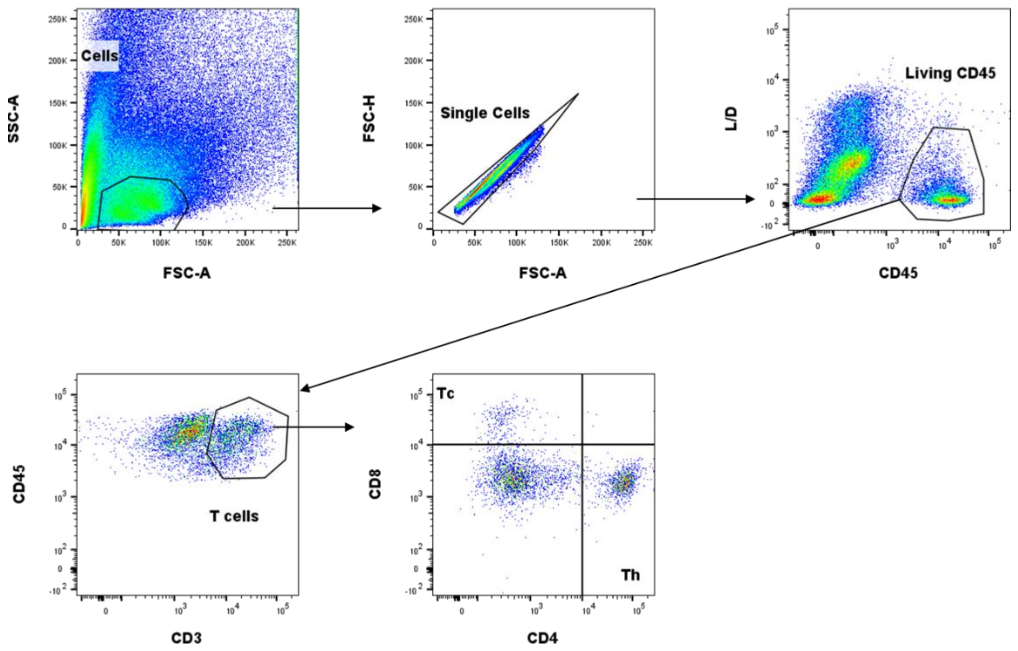

Fig 6: Flow cytometry gating strategy ($CD45^+$, $CD3^+$, $CD4^+$, $CD8^+$) of isolated pancreatic cells to differentiate T-helper (Th) and cytotoxic T-cells (Tc).
